# Global analysis of aberrant pre-mRNA splicing in glioblastoma using exon expression arrays

**DOI:** 10.1186/1471-2164-9-216

**Published:** 2008-05-12

**Authors:** Hannah C Cheung, Keith A Baggerly, Spiridon Tsavachidis, Linda L Bachinski, Valerie L Neubauer, Tamara J Nixon, Kenneth D Aldape, Gilbert J Cote, Ralf Krahe

**Affiliations:** 1 Department of Endocrine Neoplasia and Hormonal Disorders, University of Texas M. D. Anderson Cancer Center, Houston, TX, 77030, USA; 2 Graduate Program in Genes and Development, University of Texas at Houston Graduate School of Biomedical Sciences, Houston, TX, 77030, USA; 3 Department of Bioinformatics and Computational Biology, University of Texas M. D. Anderson Cancer Center, Houston, TX, 77030, USA; 4 Graduate Program in Human and Molecular Genetics, University of Texas at Houston Graduate School of Biomedical Sciences, Houston, TX, 77030, USA; 5 Department of Cancer Genetics, University of Texas M. D. Anderson Cancer Center, Houston, TX, 77030, USA; 6 Department of Pathology, University of Texas M. D. Anderson Cancer Center, Houston, TX, 77030, USA

## Abstract

**Background:**

Tumor-predominant splice isoforms were identified during comparative *in silico *sequence analysis of EST clones, suggesting that global aberrant alternative pre-mRNA splicing may be an epigenetic phenomenon in cancer. We used an exon expression array to perform an objective, genome-wide survey of glioma-specific splicing in 24 GBM and 12 nontumor brain samples. Validation studies were performed using RT-PCR on glioma cell lines, patient tumor and nontumor brain samples.

**Results:**

In total, we confirmed 14 genes with glioma-specific splicing; seven were novel events identified by the exon expression array (*A2BP1, BCAS1, CACNA1G, CLTA, KCNC2, SNCB*, and *TPD52L2*). Our data indicate that large changes (> 5-fold) in alternative splicing are infrequent in gliomagenesis (< 3% of interrogated RefSeq entries). The lack of splicing changes may derive from the small number of splicing factors observed to be aberrantly expressed.

**Conclusion:**

While we observed some tumor-specific alternative splicing, the number of genes showing exclusive tumor-specific isoforms was on the order of tens, rather than the hundreds suggested previously by *in silico *mining. Given the important role of alternative splicing in neural differentiation, there may be selective pressure to maintain a majority of splicing events in order to retain glial-like characteristics of the tumor cells.

## Background

In alternative pre-mRNA splicing, multiple transcript isoforms are expressed from a single gene by varying the combination of exons that are included in the mature mRNA. These isoforms may differ in their transcript and protein stabilities and/or in their protein structures and activities, which allows for functional and physiological diversity [1,2]. Alternative splicing affects up to 74% of all genes and may cause epigenetic instability when aberrant [3]. In cancer, two major mechanisms lead to the dysregulation of proper splicing: somatic mutations in splice regulatory *cis*-elements and mis-expression of *trans*-acting factors [4,5]. The second phenomenon has been reported in numerous cancers including glioma, ovarian and colon cancer [6-11]. Furthermore, many individual genes have cancer-predominant splicing patterns that contribute to tumorigenesis [5,12,13]. However, it is unclear whether the tumor-specific misexpression of splice factors leads to global aberrant splicing in cancer. Genome-wide attempts to address this have been performed mostly *in silico *by aligning and comparing EST libraries. Several hundred isoforms appear to be unique to tumor libraries, but these analyses are largely incomplete as they can miss known isoforms and are intrinsically biased in their scoring of single clones [14-20].

Of all tissues, the brain has the most cassette exon alternative splicing [21,22]. This tissue-specific splicing is responsible for proper neural cell differentiation and neurotransmitter signaling that may be misregulated to allow stem-cell like proliferation to form brain tumors [23-27]. Gliomas are glial-like tumors, with glioblastoma (GBM) being the most severe form [28]. Independent and *in silico *genome-wide assessments have identified genes expressing particular splice isoforms more frequently in glioma than in normal brain. Among the 27 individual (Table 1; see Additional file 1) and the five *in silico *studies (see Additional file 2), only three of the genes, *BIN1*, *MAX *and *MPZL1 *were in common. Because of these discrepancies, we performed a global, unbiased study using the human exon expression array (Affymetrix) to experimentally identify common glioma-specific aberrant splicing events and misexpressed splicing factors. Our data indicate that overall aberrant tumor-specific cassette exon splicing events involve small changes, less than 5-fold. Few splicing factors had dramatically altered expression in glioma, but could be targeting the genes that were identified as having significant glioma-specific splicing in our study.

**Table 1:** Previous individual reports of GBM-associated alternative splicing

**Gene Ontology Process**	**Genes**
Cell Growth/Apoptosis	*CDKN2B, FGFR1, MDM2, MIA, NF1, RSU-1, TP73*
Cell Mobility/Cell Adhesion	*CALD1, CD44, CRK, FN1, MAP2, ADAM22, PECAM1, TNC*
Transport	*CACNA1G, CYP27B1, SLC1A2, KCNMA1*
Transcription	*MAX, RFX4, TCF4*
Other	*FLJ12438, NRP1, SEMA6B/HAS, SR2c*

See Additional file 1 for references

## Results

### Exon array analysis

To measure significant alternative splicing changes associated with primary brain tumors, we compared genome-wide exon expression levels of 24 grade IV glioblastoma (GBM) and 12 nontumor brain samples using the Human Exon Array 1.0 ST (Affymetrix, Santa Clara, CA) [29]. Multiple statistical algorithms were developed to identify alternative pre-mRNA splicing events (see Methods). The *Differential Expression *(DE) value describes the difference in the average expression of all exons for a given RefSeq entry between two groups of samples (tumor vs. nontumor). A DE = 0 indicates no change in transcript expression between the two groups, a DE < 0 and a DE > 0 indicates decreased or increased gene expression in the disease group, respectively. The *Alternative Splicing *(AS) score was generated to identify all types of alternative pre-mRNA splicing events as detected by differential hybridization of a PSR (probe selection region). The higher the AS score for a given RefSeq entry, the greater the extent to which at least one PSR deviated in its differential hybridization compared with all other probe sets in that RefSeq. To assign a biologically relevant parameter to the AS score, we performed modeling of a cassette exon splicing event for a 3-exon gene. This gave median theoretical AS scores when comparing a 5% level of exon inclusion in one sample with a 25%, 50%, and 100% inclusion level in the second sample of 38.3, 78.7 and 132.9, respectively. As a final parameter, we included a *p*-value calculation, which indicates the probability that an AS score would show the presence of alternative splicing. We focused on a core set of RefSeq entries (20,157) with *p*-values of less than 0.05, and used the DE value plotted against the AS score to evaluate the relationship between expression and change in alternative splicing.

### Examination of FGFR1 glioma-specific splicing by exon array

**Figure 1:**
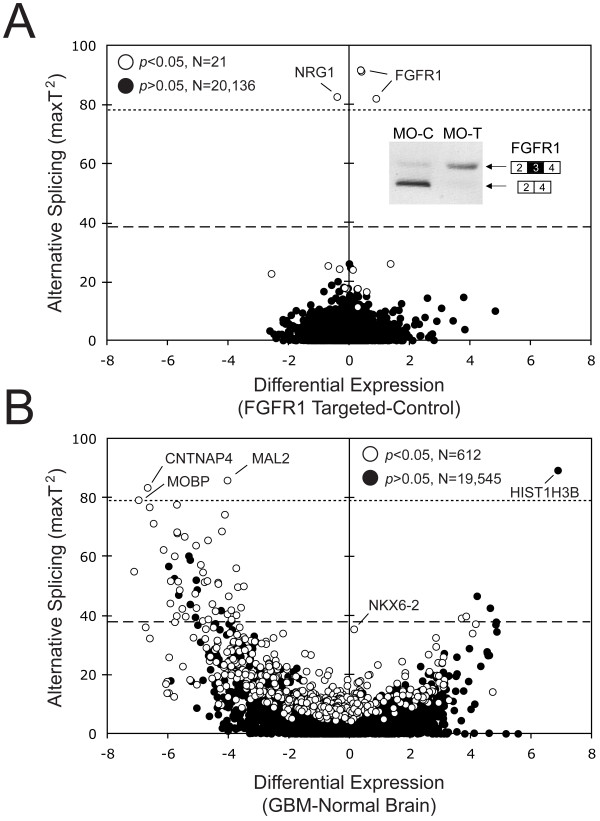
**Comparative genome-wide exon expression analyses in glioma cell lines and patient samples**. *Differential Expression *(DE) values are plotted against *Alternative Splicing *(AS) scores for a defined set of 20,157 RefSeq entries. (A) Plot showing the average of three independent experiments for U251 glioblastoma cells in which *FGFR1 *exon 3 splicing was changed using targeted antisense morpholino oligonucleotide-treatment. The positions of five RefSeq entries representing *FGFR1 *are indicated. The inset shows representative RT-PCR results for *FGFR1 *exon 3 splicing following treatment with the control (MO-C) or antisense (MO-T) oligonucleotide. For data on RefSeq entries with significant values see Additional file 4. (B) Plot showing the genome-wide changes in expression and splicing observed in GBM compared to nontumor brain. For each Ref Seq entry, the values are derived from 24 GBM samples minus 12 nontumor brain samples. Notable RefSeq entries are labeled with their gene names. For data on RefSeq entries with significant values see Additional file 5. The theoretical values for a 5-fold (dashed line) and 10-fold (dotted line) change in exon inclusion are shown. For hybridization intensity maps of the highlighted genes see Additional file 3.

In order to assess the specificity, sensitivity and feasibility of an array-based, genome-wide approach to identify alternative splice events, we determined the effect of altering a glioma-specific splicing event in U251 cells. Antisense morpholino oligonucleotides were used to switch exon 3 inclusion in *FGFR1 *mRNA precursors from its aberrant skipping to inclusion, as occurs in normal brain [30] (Figure 1A Inset). Figure 1A shows the plot of DE vs. AS values for 20,157 *core *set RefSeq entries from this exon array experiment. The *FGFR1*-specific splicing switch was easily distinguishable for five *FGFR1 *RefSeq entries that include probesets that monitor exon 3 inclusion (Figure 1A; see Additional file 3). The change in exon 3 splicing led to significant AS scores (81.99 to 91.68, *p*-value < 0.05; see Additional file 4), which were well above the median derived value for a 10-fold increase in exon inclusion. Finally, the DE values between 0.38 to 0.89 also agreed with RT-PCR results that showed little change in *FGFR1 *expression when splicing of exon 3 was altered.

The induced *FGFR1 *splicing switch also caused a large change in the splicing score of *NRG1 *(AS score of 82.57, *p*-value < 0.05). However, the hybridization map and RT-PCR validation suggest it is a cross-hybridization artifact (see Additional file 3). There were 16 other RefSeq entries, representing 11 genes that showed significant (*p*-values < 0.05) changes in exon 1 usage after treatment (see Additional file 4). These changes may be regulated by the effect of exon 3 inclusion on *FGFR1 *signaling. Overall, the U251 experiments confirmed that targeted changes in alternative splicing of cassette exons would be reflected in high AS scores and *p*-values < 0.05 on the exon array. These data indicated the general feasibility of the exon array and our analytical approach to identify cassette exon changes on a genome-wide level.

### Detection of alternative splicing in GBM patient samples

Next, we compared the genome-wide exon expression levels in 24 GBM and 12 nontumor samples (Figure 1B; see Additional file 5 for RefSeq entries with significant *p*-values < 0.05). The shape and distribution of the RefSeq values differed greatly for the patient samples compared to the *FGFR1 *experiment. Only four genes had AS scores above the derived median for a 10-fold change in splicing (*CNTNAP4*, *HIST1H3B*, *MAL2 *and *MOBP*; see Additional file 3). Unlike *FGFR1*, these genes had high levels of differential expression between nontumor and tumor samples, which appeared to impact their AS scores. *CNTNAP4 *and *HIST1H3B *are intronless genes that do not represent alternative splicing. The event in *MAL2 *involved exon 1 and could not be explained by alternative splicing. The event identified for *MOBP *was not amenable to RT-PCR verification (see Additional file 3). Finally, a significant 3' splice site event was predicted in the heat map of *NKX6-2*, which could not be confirmed by RT-PCR (Figure 1A; see Additional file 3, and data not shown).

**Figure 2:**
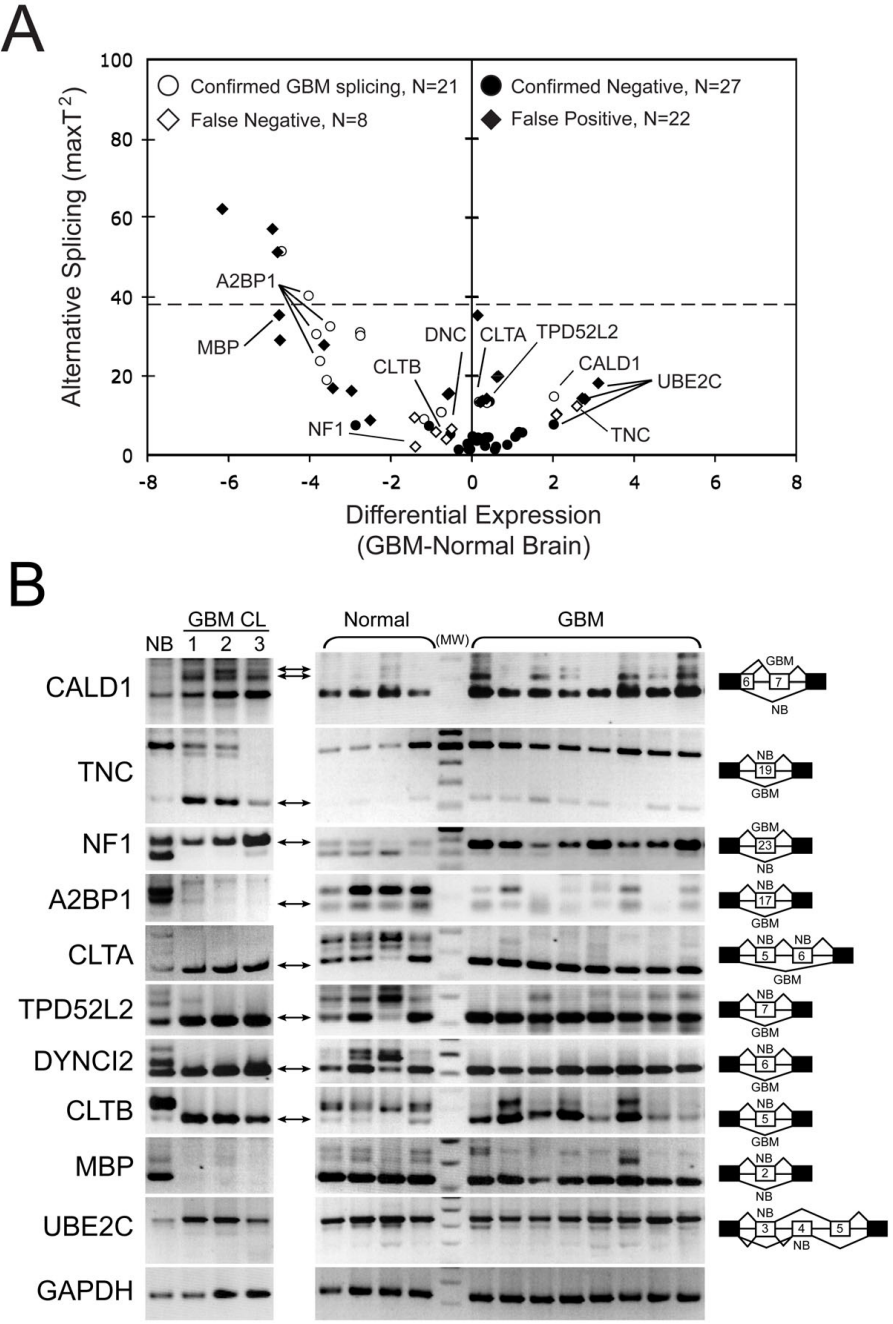
**Evaluation of glioma-specific splicing events**. (A) Plot of values extracted from Figure 1B examined by RT-PCR. False-negative samples had *p*-values > 0.05, but showed glioma-specific splicing by RT-PCR. False-positive samples had *p*-values < 0.05, but had no glioma-specific splicing by RT-PCR. Notable RefSeq entries are labeled with their gene names. The theoretical value for a 5-fold change in exon inclusion is shown (dashed line). For data on RefSeq entries with significant values see Additional file 6. (B) Representative RT-PCR validation results. The left panel shows RT-PCR results for nontumor brain (NB) and three GBM cell lines (GBM CL): U251 (1), SNB19 (2), and T98G (3). The right panel shows RT-PCR results for four nontumor brain samples and eight GBM tumor samples. The arrows indicate the isoform(s) that is differentially expressed in GBM; the involved exons are schematically presented to the right (NB, nontumor brain; GBM, GBM tumor brain). *MBP *and *UBE2C *were not observed to generate GBM-specific bands. GAPDH was used as a loading control. MW, molecular weight marker. For hybridization intensity maps for the highlighted genes see Additional file 7.

In the absence of readily identifiable large splicing changes, we went on to validate our algorithm parameters. From several hundred manually examined RefSeq entries, we chose to validate over 50 genes with hybridization heatmaps suggestive of cassette exon pre-mRNA splicing. These genes represented a broad range of AS scores, DE values, and *p*-values (Figure 2A). Validation was performed by semi-quantitative RT-PCR on three glioma cell lines (U251, SNB19 and T98G), a normal brain control, and a subset of samples from the patient set used for the array experiments (Figure 2B). We could make confident conclusions for 38 (76%) of these genes (see Additional file 6). We chose to exclude the remaining genes from our analysis due to low expression levels, which made it difficult to make definitive interpretations. The 38 genes were represented by a total of 78 RefSeq entries, with 43 entries exhibiting *p*-values < 0.05. Five entries had AS scores that were greater than the derived median for a 5-fold change in splicing. We confirmed glioma-specific splicing for eight genes represented by 21 RefSeq entries: *A2BP1*, *BCAS1*, *CACNA1G*, *CALD1*, *CLTA*, *KCNC2*, *SNCB *and *TPD52L2*. The most dramatic cassette exon changes occurred in regions where the DE values approached 0 and the AS scores were above 13 as was the case with both *CLTA *and *TPD52L2*. The lack of glioma-specific splicing was confirmed for 12 genes (26 RefSeq entries) with *p*-value > 0.05: *ALG12*, *CASP2*,*EMID1*, *FGFR1*, *PCNT2*, *LAIR1*, *MDM2*, *MNT*, *NAV2*, *PACSIN1*, *PECAM1 *and *TPM1*. Overall, RT-PCR results were concordant with array data for 47 of 78 RefSeq entries (60%). The majority of nonconcordant samples were for genes that had *p*-values < 0.05, where glioma-specific splicing could not be confirmed (false-positives; 11 genes represented by 22 RefSeq entries, or 28%). The statistical filtering missed at least six glioma-specific splicing events (false-negatives). These genes had varying degrees of glioma-specific splicing and included *APPA4, CLTB, DYNC1I2*, *NF1*, *RTN4 *and *TNC *(Figure 2; see Additional file 6). However, RT-PCR data suggested that most of these genes would have had AS scores exceeding the 5-fold threshold with the exception of *NF1 *and *CLTB*. Figure 2B shows the RT-PCR results of 10 representative genes. In total, we found 14 genes with glioma-specific splicing: *A2BP1*, *APPA4*, *BCAS1*, *CACNA1G*, *CALD1*, *CLTA*, *CLTB*, *DYNC1I2*, *KCNC2*, *NF1*, *RTN4*, *SNCB*, *TNC *and *TPD52L2*.

**Figure 3:**
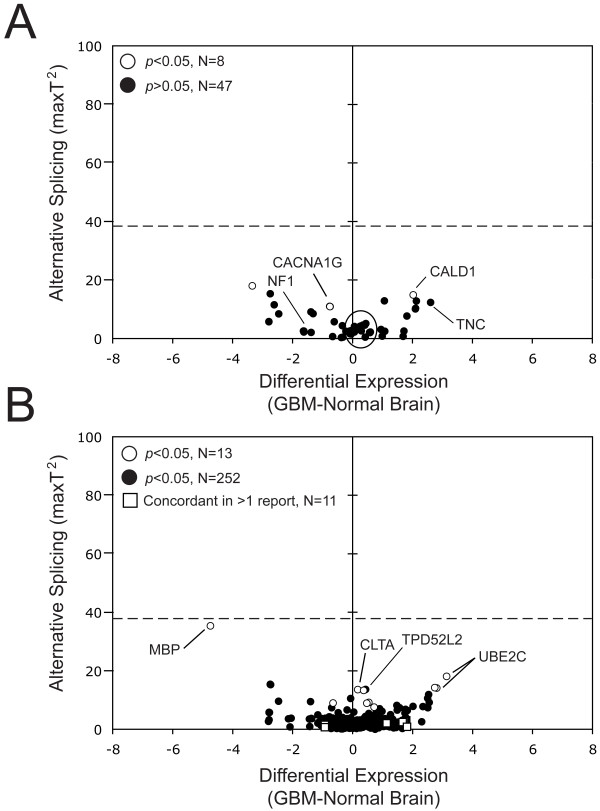
**An array-based examination of published and *in silico*-predicted glioma-specific events**. (A) Plot of values extracted from Figure 1B showing RefSeq entries that monitor published glioma-specific splicing events (Table 1). The circled RefSeq entries include confirmed negative splicing events. For data on RefSeq entries with significant values see Additional file 1. (B) Plot showing positions of 267 RefSeq entries from Figure 1B that were identified by five *in silico *studies [15–18, 20]. The open squares show 11 RefSeq events that were concordant in more than one study (discussed in the text). Notable RefSeq entries are labeled with their gene names. The theoretical value for a 5-fold change in exon inclusion is shown (dashed line). For data on RefSeq entries with significant values see Additional file 2. For the hybridization intensity maps for the highlighted genes see Additional file 8.

As an additional step to determine the accuracy of the exon array platform and our algorithm, we specifically queried previously reported GBM-specific splicing events within our dataset. Figure 3A plots the array values obtained in our patient set for 32 independently identified GBM-specific pre-mRNA splicing events (Table 1), represented by a total of 55 RefSeq entries (see Additional files 1 and 8). For all of these genes, the AS scores fell below the derived 5-fold change in splicing (the highest was 18.12 in *CAMK2A*). Only *CALD1, CACNA1G *and *CAMK2A *had *p*-values < 0.05 that predicted strong differential splicing, while *BIN1, CD44*, *RFX4 *and *TNC *had AS scores close to that of *CAMK2A*, showing small changes in splicing. Validation of a subset of these genes (*CACNA1G*, *CALD1*,*FGFR1*, *MDM2*, *NF1*, *PECAM1 *and *TNC*) by semi-quantitative RT-PCR confirmed GBM-specific splicing for four of seven (~60%) reported splicing events: *CACNA1G*, *CALD1*, *NF1 *and *TNC *(Figures 2B and 3A; see Additional file 8). *CACNA1G *had enhanced exon inclusion in the glioma cell lines, with a less pronounced change in the GBM patient samples (data not shown). The *CALD1 *differential exon inclusion event was present predominantly in glioma cell lines and GBM patient samples. For *TNC*, the GBM patient samples had more exon inclusion compared with the nontumor brain samples, which was opposite to the cell lines and previously reported findings [31]. In *NF1*, both cell line and patient samples had greatly enhanced inclusion of exon 23A, which agreed with published findings [14]. The results for *FGFR1 *were inconclusive since the glioma cell lines showed the expected predominant skipping of exon 3 compared with our normal brain control, but enhanced exon skipping was not as prominent in the patient samples. The three remaining genes (*MBD1*, *MDM2 *and *PECAM1*) had no detectable GBM-specific splicing, consistent with their low AS scores (circled in Figure 3A). Therefore, the RT-PCR analyses on these seven published events were consistent with array-derived results except for exon 23A of *NF1*, which was not represented by an established RefSeq.

### Low concordance with *in silico *studies

Previous *in silico *studies identified at least 186 genes with purported glioma-specific splicing events [15-18,20]. Figure 3B plots the array values obtained in our patient set for these genes (represented by 265 RefSeq entries; see Additional file 2). Only 13 of 265 RefSeq entries had *p*-values < 0.05 (see Additional file 8). Most of the genes had only small changes in differential expression (the majority clustered at DE = 0). Low AS scores suggest that the reported splicing events are infrequent, which could explain the lack of concordance among the studies. *CLTA *and *TPD52L2 *had clear, verifiable glioma-specific splicing patterns by both exon array and RT-PCR (Figure 2A, 2B and 3B; see Additional files 7 and 8); they were also listed in one of these reports [18]. *MBP *and *UBE2C *had *p*-values < 0.05 (Figure 2A and 3B). However, RT-PCR analysis failed to validate the presence of a consistent glioma-specific splicing event (Figure 2B).

### Expression profiling of RNA processing factors

**Figure 4:**
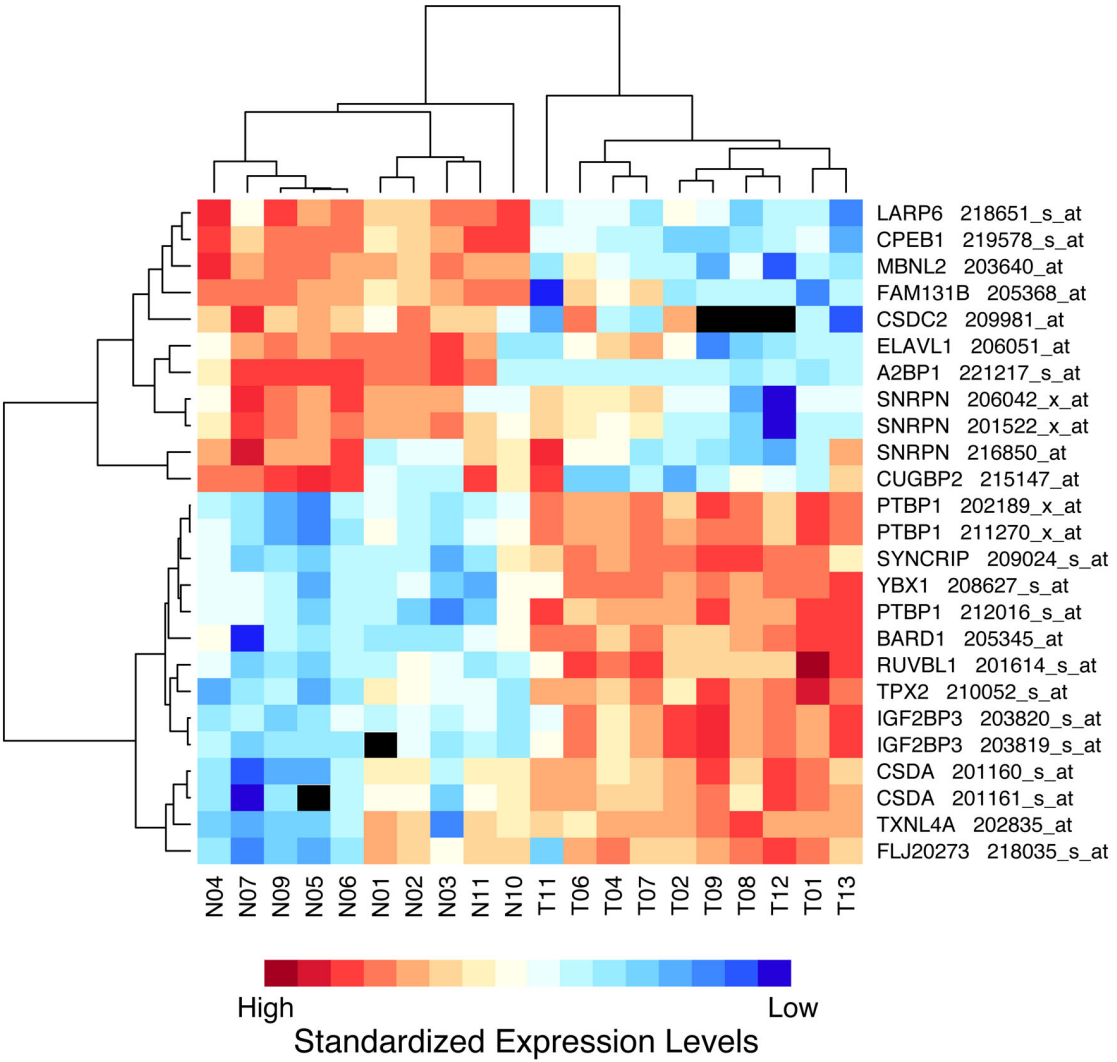
**Differential expression of RNA processing factors in GBM tumors. **Expression analysis was performed on 10 GBM tumor and 10 nontumor samples using the Affymetrix U133A Plus 2 platform. Two-way hierarchical clustering analysis of the 25 most differential probe sets with *p*-value < 0.05 is shown. For detailed information on the probe sets and their functions For the hybridization intensity maps for the highlighted genes see Additional file 10.

Recently, upregulation of the splicing factor SF2/ASF was shown to be oncogenic [11]. To determine changes in splicing factor expression, we also performed expression profiling on a subset of 10 GBM and 10 nontumor samples using the established U133 Plus 2 expression array. Overall, only 13 of 499 probe sets queried (2.6%) showed significant differences (> 4-fold, *p*-value < 0.05) in expression levels between GBM and nontumor samples (see Additional file 9). To determine whether splice factor expression could be used as a marker of gliomagenesis, we performed an unsupervised clustering analysis. In this clustering, the samples separated into GBM and nontumor groups with the exception of a single nontumor sample (data not shown). Clustering of the 25 most differentially expressed probe sets with significant *p*-values representing 19 unique genes (see Additional file 10) is shown in Figure 4. The majority of these genes are not known to be associated with alternative splicing. However, three splice factors could be linked to GBM-specific splicing events having *p *< 0.05 (see Additional file 9): A2BP1 regulates *CLTB*, *GRIN1*, *MAG*, *NF1 *and *SCN8A *splicing, PTB regulates *CLTB*, *GABA*, *GRIN *and *FGFR2 *splicing, and CUGBP2 regulates *RASGRF1 *splicing [32-35].

## Discussion

Aberrant pre-mRNA splicing may be an important epigenetic factor for tumor progression. However, it is unclear how many genes are mis-spliced in a given tumor and whether aberrant expression of splice factors is responsible for their appearance. We used an exon array and designed analytical algorithms and parameters to identify GBM-specific splicing events in an unbiased manner. By plotting scores for differential expression (DE) against those for alternative splicing (AS) for genes and exons interrogated by the exon array, we were able to distinguish a single targeted induced splicing change in *FGFR1 *among 20,157 RefSeq entries and to monitor the concomitant splicing and gene expression changes. Using the same approach for comparing an extended GBM and nontumor sample set, GBM-specific splicing events of similar magnitude were not identified, which suggests that large-scale aberrant splicing in GBM are infrequent. We do not discount the fact that individual instances of dramatic changes in splicing were present; however, they were not shared by the majority of samples. The lack of events with large changes in differential exon expression led us to mine our data for splicing changes with at least a 2-fold change in probe set hybridization (AS score) and a *p*-value < 0.05. In the hundreds of heatmaps examined, there were many changes indicative of the usage of alternative promoters or polyadenylation sites. However, we chose to focus on cassette exons as events that could be readily examined by RT-PCR. This led to the validation of 14 GBM-specific events: *A2BP1*, *APPA4*, *BCAS1*, *CACNA1G*, *CALD1*, *CLTA*,*CLTB*, *DYNC1I2*, *KCNC2*, *NF1*, *RTN4*, *SNCB*, *TNC *and *TPD52L2*. Moreover, our expression profiling analysis indicated that there were relatively few GBM-specific changes for splicing regulators. Among the genes with the greatest differential expression only *A2BP1*, *CUGBP2*, *ELAV1*, *MBNL2*, *PTBP1 *and *YBX1*, have known functions in alternative splicing. At least three of these (*A2BP1*, *PTBP1 *and *CUGBP2*) could be linked to GBM-specific splicing events.

The identified and validated GBM-specific isoforms encode proteins that primarily affect cell growth and mobility. A2BP1, which shows both differential expression and splicing, is a neuronal-specific splicing regulator for multiple targets [36]. CLTB and DYNC1I2 are involved in intracellular transport and may play a role in cell migration [37,38]. Four genes have clearly identified functions in the central nervous system: APPA4 functions in Notch signaling during neural development, cell adhesion and apoptosis; RTN4 is a neurite growth inhibitor; and SNCB, which is upregulated in glial tumors, is thought to regulate phospholipase D2 activity. NF1 is believed to be a glial-cell marker and mutated in multiple CNS tumors [14,39]. For the remaining genes, little is known about their function in normal brain or gliomagenesis. Comparing glioblastoma and oligodendroglioma as two histological glioma subgroups on the same exon array platform that we used here, French and colleagues recently identified a total of 11 differentially expressed splice variants [40], one of which overlapped with our validated genes (*CAMK2A*) (see Additional file 1). In the exon array study for prostate and colon cancer, only *CALD1 *was in common with our validated gene list [29,33]. Therefore, it is unclear whether common pathways are targeted for splicing changes during tumorigenesis. It remains to be determined whether these splicing targets have a synergistic effect on gliomagenesis.

Genes with glioma-predominant splice isoforms have previously been identified through global EST alignments. *MAX *was the only gene found both experimentally and *in silico *(see Additional files 1 and 2). Despite using similar datasets, the number of *in silico *derived genes with GBM-specific isoforms varied and only three genes were found to be shared between two of the five studies: *AP2A1*, *CPNE1 *and *KPNB1 *[14,15,17,26,27]. The lack of agreement between all of these studies can be explained by several technical and biological factors. First, the small sample sizes used did not allow for statistical calculations. Second, sample heterogeneity affected normalization and interpretation. Third, the nature of the samples being compared, which can be normal or nontumor *vs*. tumor, or tumor type A *vs*. tumor type B. For EST libraries, the true splicing frequency could be masked by too few ESTs, normalization strategies, and/or low-stringency criteria that enriched for rare ESTs [41,42]. The bias towards 3' and 5' ends of transcripts could also lead to the under-representation of isoforms with internal differences [41,42]. In contrast, using a large sample set on exon arrays circumvented these problems and allowed for objective measurements of isoform frequencies. It should be noted, however, that array-based studies are limited by the quality of the target preparation, probe specificity and sensitivity, and for the Affymetrix platform we used the lack of interrogation of exons < 25 bp., and can be confounded by tumor tissue heterogeneity. Of our 14 validated GBM-associated splicing events, eight (*CAMK2A*,*CACNA1G*, *CALD1*, *CLTA*, *NF1*,*RTN4*, *TNC *and *TPD52L2*) were previously reported (see Additional files 1 and 2). Most of the genes that were discordant had splicing changes that were outside the range of detection for the array (less than 2-fold). Two additional genes (*MBP *and *UBE2C*) had a *p*-value < 0.05 that were concordant with *in silico *determined genes, but could not be validated by RT-PCR (Figure 2B). Finally, the lack of complete agreement in all of these gene lists could be due to the overall low level of aberrant splicing in GBM.

Many studies have shown that overexpression of a single cancer-enhancing isoform is sufficient to alter glioma cell proliferation or invasion [43-45]. What remains unclear is how these specific isoforms are produced. Many cancers have over- or under-expression of splicing factors, which suggests that global aberrant splice events are possible. However, our analysis revealed that aberrant splicing factor expression does not lead to either large changes in specific exon utilization or widespread changes in the splicing of multiple targets. It is likely that titration of levels of multiple splicing factors is required to "fine tune" splicing decisions.

## Conclusion

The relatively small number of aberrant pre-mRNA splicing events that we observed in our GBM sample set suggests that systematic, epigenetic targeting of splicing that leads to large changes may not be an important mechanism in gliomagenesis. Other exon array studies measuring tumor-specific aberrant alternative pre-mRNA splicing in prostate cancer and colon cancer also support the finding that extreme splicing changes in cancer are less frequent than was suggested by *in silico *studies [29,33,40,46]. We found that only 612 of 20,157 (3%) fully annotated RefSeq entries on the exon array showed significant changes in exon expression. We interpret our results to indicate that aberrant pre-mRNA splicing in GBM is a low frequency event. However, this analysis does not rule out patient- or gene-specific aberrant splicing events, or smaller magnitude splicing changes with critical functions in gliomagenesis. Furthermore, the differential expression of several RNA processing factors not involved in alternative splicing suggests that other aspects of RNA biology may play critical roles in gliomagenesis. Our validation experiments indicate that GBM-specific splicing is generally a partial event, with varying degrees of exon inclusion. The 14 genes identified in our study are potentially the most important GBM-specific splicing events and constitute promising targets for therapeutic intervention.

## Methods

### Cell line information

The U251, SNB19 and T98G cell lines were grown as previously described [30]. Morpholino oligonucleotides targeting the intron splicing silencer sequences flanking *FGFR1 *exon 3 was performed by double "scrape-loading" as previously described [30]. RNA isolation was performed using the RNeasy Micro kit according to the instructions provided (Qiagen, Valencia, CA).

### Patient information

Informed consent was obtained from all patients prior to sample collection in accordance with the guidelines set by the Institutional Review Board. Biopsy samples from surgical resections were collected and banked through the brain tumor tissue bank at the Brain Tumor Center at the University of Texas M. D. Anderson Cancer Center. All samples were immediately placed on ice and snap-frozen for -80°C storage within 30 min of devascularization after removal of portions needed for pathological diagnosis. We obtained 24 newly diagnosed primary glioblastoma (WHO grade IV astrocytoma; K. Aldape) samples from patients who received no therapy, but underwent gross total resection before sample collection. Glioblastoma samples contained at least 90% tumor. 12 frozen samples of nonneoplastic brain tissue without histologic evidence of tumor or another significant abnormality were used for comparison. RT-PCR validation also included Human Brain Total RNA, extracted from a 78-year-old Caucasian female with congestive heart failure (Ambion, Austin, TX).

### RNA extraction

RNA was extracted using the TriZol Reagent according to the manufacturer's suggestions (Invitrogen, Carlsbad, CA) and further purified using the RNeasy kit. The quality and integrity of the RNA was then analyzed on an Agilent BioAnalyzer (RNA 6000 Nano LabChip). Total cellular RNA samples with a RIN (RNA integrity number) > 7 were used for further microarray studies.

### Microarray exon profiling

For exon profiling on the Human Exon Array 1.0 ST (Affymetrix Santa Clara, CA), RNA was prepared using a pre-commercial versions of the Affymetrix GeneChip Whole Transcript (WT) Double-Stranded Target Labeling Assay for preparation of double-stranded (ds) target DNA (first generation protocol) or the Affymetrix GeneChip Whole Transcript (WT) Sense Target Labeling Assay for preparation of single-stranded (ss) target DNA (second generation protocol). Precommercial versions of the kits, containing identical formulations to the commercial kits, were used for target preparation. For comparison a subset of samples was prepared with both protocols; however, samples prepared with the different protocols were not mixed in subsequent analyses.

#### Double-stranded target preparation

100 ng of total RNA was added to a solution of 250 ng/μl T7-(N)6 primer/poly(A) RNA control dilution for a total volume of 5 μl. Samples were mixed and incubated at 70°C for 5 min and cooled at 4°C for 2 min. A first-cycle, first strand cDNA synthesis mix was prepared by combining 2 μl of 5× first-strand buffer, 1 μl of 0.1 M DTT, 0.5 μl of 10 mM dNTP mix, 0.5 μl of 40 U/μl RNase-Out and 1 μl of 200 U/μl SuperScript II. The first-cycle, first strand cDNA synthesis mix was added to the total RNA T7-(N)6 primer/poly(A) RNA control for a total volume of 10 μl, mixed, centrifuged and incubated at 25°C for 10 min, 42°C for 60 min, 70°C for 10 min and 4°C for 2 min. A first-cycle, secondstrand cDNA synthesis was then prepared using 4 μl of 17.5 mM MgCl2, 0.4 μl of 10 mM dNTP Mix, 2.5μl of 5 U/μl Klenow 3' to 5' exo, 0.2 μl of 2 U/μl RNase H and 2.9 μl nuclease-free water. Ten μl from the first-cycle, first strand cDNA synthesis reaction was added for a total volume of 20 μl, mixed, centrifuged and incubated at 37°C for 50 min, 75°C for 10 min, and 4°C for 2 min. Using components from the MEGAscript T7 Kit (Ambion, Austin, TX), 5 μl of 10× reaction buffer, 5 μl ATP, 5 μl CTP, 5 μl UTP, 5 μl GTP and 5 μl enzyme mix were combined with each reaction for a total volume of 50 μl. Reactions were incubated at 37°C for 16 hrs. Reactions were then immediately cleaned using the cRNA Cleanup Spin Columns from the GeneChip Sample Cleanup Module (Affymetrix). One μl of the sample was used for cRNA yield determination on the spectrophotometer; 15 μg of cRNA was mixed with 9 μg of random primers and nuclease free water for a reaction volume of 24 μl. Reactions were incubated at 70°C for 5 min, 25°C for 5 min and 4°C for 2 min. A second-cycle, first-strand mix was prepared using 12 μl 5× first-strand buffer, 6 μl of 0.1 M DTT, 3 μl of 10 mM dNTP mix with dUTP (Affymetrix), 3 μl of 40 U/μl RNase-Out and 12 μl of 200 U/μl SuperScript II; 36 μl of the second-cycle, firststrand mix was added to the cRNA and random primers, mixed, centrifuged (60 μl total) and each reaction was divided into 3 tubes of 20 μl. Reactions were incubated at 25°C for 5 min, 42°C for 60 min, 70°C for 10 min, 4°C for 2 min. A second-cycle, second-strand cDNA synthesis solution was prepared using 24 μl of 17.5 mM MgCl_2_, 1.8 μl of 10 mM dNTP mix with dUTP (Affymetrix), 16.2 μl of 5 U/μl Klenow 3' to 5' exo, 1.5 μl of 2 U/μl RNase H and 16.5 μl nuclease-free water. The three 20 μl reactions were combined into one tube and 60 μl of secondcycle, second-strand cDNA synthesis solution was added for a total of 120 μl. Reactions were mixed, centrifuged and the samples were divided again into 3 tubes of 40 μl each. Reactions were incubated at 37°C for 50 min, 75°C for 10 min and 4°C for 2 min. Reactions were then cleaned using the cDNA Cleanup Spin Columns from the GeneChip Sample Cleanup Module (Affymetrix). Once cleaned the three tubes were combined into one, mixed and measured on a spectrophotometer. Twenty μg of double stranded (ds) cDNA (up to 96.6 μl) was fragmented with 14.4 μl of 10× NEB Buffer 4, 12 μl of 2 U/μl uracil DNA glycosylase and 21 μl of 10 U/μl APE 1. Reactions were mixed, centrifuged and then split into 3 tubes (48 μl each), incubated at 37°C for 60 min, 93°C for 1 min and 4°C for 2 min. One μl was removed from each tube for analysis on the Agilent BioAnalyzer using the RNA 6000 Nano LabChip. The three tubes were combined again (up to 135 μl), and labeled with 36 μl of 5× TdT Reaction Buffer, 6 μl of 30 U/μl rTDT and 3 μl of GeneChip DNA Labeling Reagent DLR (Affymetrix). Samples were mixed, centrifuged, divided 60 μl per tube and incubated at 37°C for 60 min and 4°C for 2 min. The reactions were stopped by adding 2 μl of 0.5 M EDTA, combined into one tube and concentrated using the YM-3 Microcon column.

#### Single-stranded target preparation

One μg of RNA was mixed with diluted (1:20, 1:50, 1:50) Affymetrix poly(A) controls (2 μl) and a Ribo-Minus probe (Invitrogen). The RNA was then ribo-reduced using the Ribo-Minus Human/Mouse Transcriptome Isolation Kit (Invitrogen). To dilute the Ribo-Minus probe, hybridization buffer was prepared by combining 45 μl of 5 M betaine and 105 μl of Invitrogen hybridization buffer; 0.8 μl of the Ribo-Minus probe (100 pmol/μl) was diluted with 30 μl hybridization buffer containing betaine and then added to the RNA and poly(A) control mix. Reactions were incubated at 70°C for 5 min. Magnetic beads used for the ribo-reduction step were washed twice with RNase-free water, once with hybridization buffer with betaine and resuspended with 20 μl of hybridization buffer with betaine. The magnetic beads were warmed at 37°C for 2 min prior to the addition of the RNA poly(A) control probe. Following the 70°C incubation, the samples were immediately cooled on ice for 2 min. Samples were then added to the magnetic beads, mixed, centrifuged and incubated at 37°C for 10 min with a mix after 5 min. Following incubation the samples were placed on a magnetic stand for 5 min, and the supernatant was removed and placed on ice. The beads were washed with hybridization buffer and betaine and incubated at 50°C for 5 min. The samples were then placed back on the magnetic stand for 5 min, and the supernatant was removed and placed on ice. The rRNA-reduced RNA was concentrated using the GeneChip IVT cRNA Cleanup Kit (Affymetrix). One μl of concentrated RNA was analyzed on an Agilent BioAnalyzer using the RNA 6000 Nano LabChip to check the quality and percent of ribosomal reduction of the starting material. Percent ribosomal reduction of the RNA ranged between 60–90%. Four μl of ribosomal reduced RNA was added to 500 ng/μl of T7-(n)6 primers for a total of 5 μl and incubated for 5 min at 70°C, 5 min at 25°C and cooled for 2 min at 4°C. Using components from the GeneChip WT Double Stranded cDNA Synthesis Module (Affymetrix) 2 μl of 5× 1st strand buffer, 1 μl of DTT 0.1 M, 0.5 μl of dNTP 10 mM, 0.5 μl of RNase Inhibitor and 1 μl SuperScript II 200 U/μl) were combined and added to the concentrated rRNA-Reduced total RNA/poly(A) RNA controls/T7-(N)6 primers for a total of 10 μl. Reactions were incubated at 25°C for 10 min, 42°C for 60 min, 70°C for 10 min, 4°C for 2 min. A first-cycle, second-strand solution was then prepared again using the GeneChip WT Double Stranded cDNA Synthesis Module and added to each sample (4 μl of 17.5 mM MgCl2, 0.4 μl of 10 mM dNTP mix, 0.6 μl of DNA polymerase I, 0.2 μl of RNase H, and RNase-free water for a total reaction volume of 20 μl). Reactions were mixed and incubated at 16°C for 2 hrs, 75°C for 10 min and 4°C for 2 min. An IVT solution was prepared using the GeneChip WT cDNA Amplification Kit (Affymetrix; 5 μl of 10× IVT buffer, 20 μl of IVT NTP mix and 5 μl of IVT Enzyme mix), added to each reaction and allowed to incubate at 37°C for 16 hrs. Reactions were immediately cleaned up using the cRNA Cleanup Spin Columns from the GeneChip Sample Cleanup Module (Affymetrix). The second cycle, first-strand cDNA synthesis was prepared by adding 8 μg of cRNA with 4.5 μg of random primer and RNAse-free water for a total volume of 8 μl and incubated at 70°C for 5 min, 25°C for 5 min, and cooled at 4°C for 2 min. The second-cycle reverse transcription mix was prepared using the GeneChip WT cDNA Amplification Kit (Affymterix) by combining 4 μl 5× 1st Strand Buffer, 2 μl DTT 0.1 M, 1.25 μl 10 mM dNTP+dUTP 10 mM, and 4.75 μl of SuperScript II 200 U/μl. This was immediately added to the second-cycle, first-strand cDNA synthesis upon cooling. Reactions were then mixed and incubated at 25°C for 10 min, 42°C for 90 min, 70°C for 10 min, 4°C for 2 min. One μl of RNase H was then added to each sample for hydrolysis of the dsDNA and incubated at 37°C for 45 min, 95°C for 5 min and 4°C for 2 min. Reactions were then cleaned using the cDNA Cleanup Spin Columns from the GeneChip Sample Cleanup Module. Five μg of single-stranded (ss) DNA was then fragmented and labeled using the Gene Chip WT Double Stranded DNA Terminal Labeling Kit (Affymetrix; 4.8 μl of 10× cDNA Fragmentation Buffer, 1 μl of 10 U/μl of UDG, and 1 μl of 1000 U/μl of APE 1). Samples were mixed and incubated at 37°C for 60 min, 93°C for 2 min and 4°C for 2 min. Two μl of this reaction was saved for analysis on the Agilent BioAnalyzer using the RNA 6000 Nano LabChip. The remainder of the sample was labeled with 12 μl of 5× TdT Buffer, 2 μl of TdT and 1 μl of 5 mM DNA labeling reagent for a total volume of 60 μl. Reactions were mixed and incubated at 37°C for 60 min, 70°C for 10 min and 4°C for 2 min. Five μg of either dsDNA or ssDNA target preparations were hybridized with 50 pM oligonucleotide B2, 20× Eukaryotic Hybridization Controls (bioB, bioC, bioD, cre) at 1.5, 5, 5 and 100 pm, 0.1 mg/ml herring sperm DNA, 0.5 mg/ml acetylated BSA, 1× hybridization buffer, 7% DMSO and up to 220 μl RNase-free water. Reactions were mixed and briefly centrifuged then incubated at 99°C for 5 min, cooled to 45°C for 5 min and then centrifuged at maximum speed for 1 min to collect precipitate. Exon arrays were equilibrated to room temperature prior to injection; 200 μl of the hybridization solution was added to each array and incubated in a rotating hybridization oven (Affymetrix) at 45°C and 60 rpm for 16 hrs. Following hybridization, the arrays were washed and stained on an Affymetrix Fluidics 450 workstation using the FS450_0001 fluidics script, and scanned on an Affymetrix GeneChip 3000. GeneChip Operating Software (GCAS) v1.3 was used to produce .cel intensity files.

### Exon array data analysis

Exon arrays were quantified using the PLIER algorithm introduced by Affymetrix. The arrays were quantile-normalized, and GC-specific background was estimated and subtracted using the PM-GCBG option. All quantifications used log_2_(PLIER + 8) values, where the value "8" is an arbitrary shrinkage constant. PLIER summarizes groups of probe-level intensities, and we used two different groupings: (1) all probes within a single PSR, and (2) all probes within a given gene. Genes were defined as RefSeq clusters using groupings of PSRs supplied by Affymetrix. While many PSRs on the array are not part of a RefSeq cluster, we chose to focus primarily on those where we had good annotation. Exon numbering within the gene was checked by mapping the reported sequences against data from the UCSC genome browser (Build 16).

#### Alternative splicing

In order to identify alternative splicing within a gene, we determined instances where the PSR-level quantifications differed significantly from the gene-level quantifications. For example, let us consider the case of a hypothetical gene with several component PSRs (Gene A). The gene-level PLIER quantifications suggest mean log_2 _intensities of 8 in GBMs and 7 in nontumor brain (a two-fold increase in expression in GBMs). Quantifications of the first PSR suggest mean values of 7 in GBMs and 6 in nontumor brain, again showing a two-fold increase in the GBMs, consistent with the overall behavior of the gene. Taking the mean difference for the PSR (7 - 6 = 1), we subtract the mean difference for the gene (8 - 7 = 1) to get the amount by which the behavior of the PSR differs from that of the overall gene; large differences specific to the PSR indicate potential alternative splicing. While "large" can be assessed in terms of fold-change, we prefer to scale the observed (PSR change) – (gene change) by an estimate of the within group (GBM or nontumor) standard deviation, as in a standard *t*-test. For example, let us assume that these differences for one PSR have values of (0 - 2, 0 - 1, 0, 0 + 1, 0 + 2) for the GBM and (2 - 2, 2 - 1, 2, 2 + 1, 2 + 2) for the nontumor samples. The mean difference between groups is 2. Now let us assume that for another PSR we see (0 - 6, 0 - 3, 0, 0 + 3, 0 + 6) *vs*. (2 - 6, 2 - 3, 2, 2 + 3, 2 + 6). The mean difference (on a log_2 _scale) is still 2, but the variability is a lot higher. The latter PSR would get a smaller *t*-value than the former, since the signal does not stand out as clearly above the noise. This approach is very similar to starting with a standard two-way Analysis of Variance (ANOVA) with main effects for condition (GBM or nontumor) and PSR, and testing for a significant interaction between condition and PSR. There, the scaled PSR – gene differences are squared and summed to give the test statistic. However, since we are looking for differences in cassette exons (a very small number of PSRs), and summing spreads the differences across several PSRs, we decided to work first with the squared terms for each individual PSR and look at the size of the largest of these terms [the maxT^2^, which is our *Alternative Splicing *(AS) score]. This, in turn, is similar to the ANalysis of Splice Variation (ANOSVA [47]) save that separate variance estimates are computed for each PSR. This focus on PSR-specific variation also differs from the correlation-based method used by French *et al*. [40]. We estimated the null distribution of this statistic through simulations. Some of the PSRs found using this approach were driven by a combination of "dead probes" and gene-level differential expression: e.g., the mean level of gene expression went from 6 in the GBMs to 9 in the nontumor, but the values for one PSR stayed fixed at 3 throughout. While this PSR behaves differently from the rest of the gene, it may be doing so because the probes simply fail to hybridize to the target at all. In order to address this problem, we added a further filtration step. First, every PSR was assessed a prior probability of being measurable by taking the PLIER values across all samples from PSRs that were part of a RefSeq gene, forming a histogram, and modeling these expression values as coming from a mixture of a normal distribution (noise) and an exponential distribution (signal). Then, using the estimated parameters of the normal distribution, the prior probability with mean intensity × is normcdf(x, mu+3 * sigma, sigma^2^). PSRs close to the noise level are not likely to be measurable. Based on the observed data, this prior is updated to give a posterior distribution suggesting whether the PSR is measurable. If the PSR shows reasonable variation or difference in expression between conditions, the posterior probability of measurability should be near certainty. Updating was performed by computing *p*-values for PSR specific *t*-tests without adjusting for gene-level differences; the associated *p*-values were then used to compute posterior odds using the method of Sellke *et al*. [48]. We then computed *p*-values (using the conservative assumption that the maxT^2 ^distribution was the maximal order statistic from k independent tests) for each inclusion/exclusion combination, *i.e*., there are 2^k-k-1 ^possible combinations of "include this PSR, exclude that one" for defining a gene if we omit the null cases of 0 or 1 PSRs from consideration. For each such combination, we compute a T^2 ^value and *p*-value as noted above, and we also assign a weight proportional to the product of the probabilities of inclusion (exclusion) used. The weighted sum of these is the *p*-value reported here. In the case of the "dead" PSR discussed above, the terms with that PSR included would have small *p*-values (it is different from all of the others), but low weight (because that PSR is deemed not measurable), and the final weighted *p*-value will be large.

We also determined modal values of the test statistic via simulation. Our simulations assumed the "ideal case." The mean expression levels for individual PSRs were chosen from the interval [3,12], reflecting the range of log_2 _PLIER scores observed; normal noise was added. Variability was allowed to be different for each PSR, with the variance drawn at random from the set of PSR variances observed in our studies. Pooled variance estimates were used. Differences were concentrated in a single PSR, with the size of the difference being log_2 _of 5, 10, or 20, respectively. A difference of 0 was also examined to supply estimates of the null distribution. Simulations were run for genes involving 4, 8 and 20 exons.

#### Differential expression

Differential expression at the gene level was assessed using 2-sample *t*-tests. This is our measure of *Differential Expression *(DE). We corrected for multiple testing by using beta-uniform mixture (BUM) models [49] and targeting a false-discovery rate (FDR) of 5%. An arbitrary constant (0.1) was added to the pooled variance estimates before the t-statistics were computed to ensure a certain minimal fold-change that was statistically significant.

### Microarray expression profiling

#### Target preparation

For RNA expression profiling on the U133 Plus 2 GeneChip (Affymetrix, Santa Clara, CA), a total of 5 μg of total cellular RNA from each sample was used for cDNA synthesis according to the manufacturer's protocol. Briefly, a mixture of *in vitro *transcribed cRNAs of cloned bacterial genes for *lysA*, *pheB*, *thrB*, and *dap *(American Type Culture Collection) was added as external controls to monitor the efficiency of cRNA synthesis. First-strand cDNA synthesis was performed at 42°C for 1 hr with the Superscript II system (GIBCO/BRL) at a final concentration of 1× first-strand synthesis buffer, 10 mM DTT, 500 μM dNTPs, 100 pmol of T7-(T)_24 _primer, and 200 units of reverse transcriptase. Second-strand cDNA synthesis was performed at 16°C for 2 hr at a final concentration of 1× second-strand buffer, 250 μM dNTP, 65 U/ml DNA ligase, 250 units/ml DNA polymerase I, 13 U/ml *RNase *H. Second-strand synthesis reaction mixtures were cleaned up with an Affymetrix cDNA purication column. *In vitro *transcription labelling with biotinylated UTP and CTP was performed according to the manufacturer's recommendations (Enzo Diagnostics) for 16 hr at 37°C. Amplified cRNA was purified on a cRNA purification column (RNeasy, Qiagen), and the quality of the amplification was verified by analysis on an Agilent BioAnalyzer. Labelled cRNAs were fragmented for 35 min at 94°C in 40 mM Tris-acetate, pH 8.1/100 mM KOAc/30 mM Mg(OAc)_2_. The hybridization cocktail consisted of 10 μg fragmented cRNA in 200 μl, containing 50 pM control oligonucleotide B2, 0.1 mg/ml herring sperm DNA, 0.5 mg/ml acetylated BSA, 100 mM Mes, 20 mM EDTA, 0.01% Tween 20 (total Na^+ ^= 1 M), and bacterial sense cRNA controls for *bioB*, *bioC*, *bioD*, and *cre *at 1.5, 5.0, 25, and 100 pM, respectively. Fragmented cRNAs were then hybridized to Affymetrix U133Plus2 GeneChips and scanned according to the manufaturer's protocol.

#### Expression array data analysis

For the U133 Plus 2.0 expression array, we identified a comprehensive set of 499 probe sets representing RNA processing factors (RPFs) with known functions in general and alternative splicing, RNA export, RNA degradation, miRNA processing and nonsense-mediated decay (see Additional file 9). Gene-level RMA quantifications were used for unsupervised clustering analysis on the 499 probe sets. To identify the most relevant RPFs, we performed two-sample *t*-tests for each gene to contrast the groups revealed by the clustering analysis. Values with a *p*-value < 0.05 were extracted and sorted by the difference in mean expression between the two groups. Values for the top 25 positive or negative mean expression differences were then used to perform a second clustering analysis shown in Figure 4.

### RT-PCR validations

cDNA was generated from 2 μg of *DNase*I treated brain, tumor, or cell line RNA using standard methods. Equal amounts of random decamer and oligo-(dT) primed cDNA were pooled and diluted to 200 μl with molecular grade H_2_O. For PCR, 5 μl for patient samples and 3 μl for glioma cell lines of cDNA were used. PCR was carried out in 1× PCR buffer (Invitrogen, Carlsbad, USA), 1.5 mM MgCl_2_, 0.25 mM dNTPs, 20 pmol of each forward and reverse primer (for primers see Additional file 111), and 2.5 U of Taq Polymerase (Invitrogen, Carlsbad, USA). The reaction was carried out for 35–37 cycles at 55–62°C annealing depending on the primer set and transcript abundance. The PCR products were electrophoresed on 1% agarose gels and visualized with ethidium bromide staining.

## Authors' contributions

HCC performed RT-PCR validations and data analysis, reviewed previously reported data and drafted the manuscript. KAB formulated the algorithms used for the exon array data analyses, performed the clustering analyses on RNA processing factors, supervised all statistical analyses, and wrote the statistical sections of the manuscript. ST performed statistical analyses of exon array data and generated data tables. LLB contributed to experimental design, data interpretation, and writing of the draft manuscript. VLN and TJN performed all exon array experiments. KDA ascertained the patient samples and their clinical information. GJC and RK designed the experiments, interpreted exon array data and analyses, performed the RNA processing factor analysis, and wrote the final manuscript. RK supervised the overall project. All authors read and approved the final manuscript.

## Supplementary Material

Additional file 1 **Published genes reported to have glioma-specific splicing that were plotted in **Figure 3A. Gene Information, T-values, *p*-values for published genes for Figure 3A. Click here for file

Additional file 2 ***In silico *genes in **Figure 3B. Gene Information, T-values, *p*-values for *in silico *genes for Figure 3B. Click here for file

Additional file 3 **Hybridization intensity maps for genes identified in **Figure 1A and 1B. Gene Information, T-values, *p*-values for *in silico *genes for Figure 3B. Click here for file

Additional file 4 **RefSeq entries with *p*-values < 0.05 for **Figure 1A. Gene Information, T-values, significant *p*-values for U251 cells treated with FGFR1 antisense morpholino oligonucleotides for Figure 1A. Click here for file

Additional file 5 **RefSeq entries with *p*-values < 0.05 in the normal brain versus glioma patient samples comparison shown in **Figure 1B. Gene Information, T-values, significant *p*-values for NB vs. GBM data for Figure 1B. Click here for file

Additional file 6 **Data for RT-PCR validations shown in **Figure 2A. Gene Information, T-values, *p*-values for RT-PCR-tested genes for Figure 2. Click here for file

Additional file 7 **Hybridization intensity maps for genes identified in **Figure 2. Heat Maps for Figure 2. Click here for file

Additional file 8 **Hybridization intensity maps for those genes identified in **Figure 3A and 3B. Heat Maps for Figure 3. Click here for file

Additional file 9 **Differential expression of RNA processing factors between GBM and nontumor samples**. Gene Information, Probeset IDs, Expression-values, and *p*-values for all genes identified as associated with RNA processing. Click here for file

Additional file 10 **Top 25 differential probesets with significant differences between the two major clustered groups shown in **Figure 4. Gene Information, Probeset IDs, Expression-values, and *p*-values for genes identified in Figure 4. Click here for file

Additional file 11 **Primers used in RT-PCR validations**. List of Primers used for RT-PCR reactions. Click here for file
