# Supplementary material for: Global analysis of aberrant pre-mRNA splicing in glioblastoma using exon expression arrays
Source: BMC Genomics. 2008 May 12;9:216. doi: 10.1186/1471-2164-9-216 (PMC2410136; doi:10.1186/1471-2164-9-216)
Supplement: Additional file 3 — Hybridization intensity maps for genes identified in Figure 1A and 1B. Gene Information, T-values, p-values for in silico genes for Figure 3B. [file 1471-2164-9-216-S3.ppt]

## Slide 1
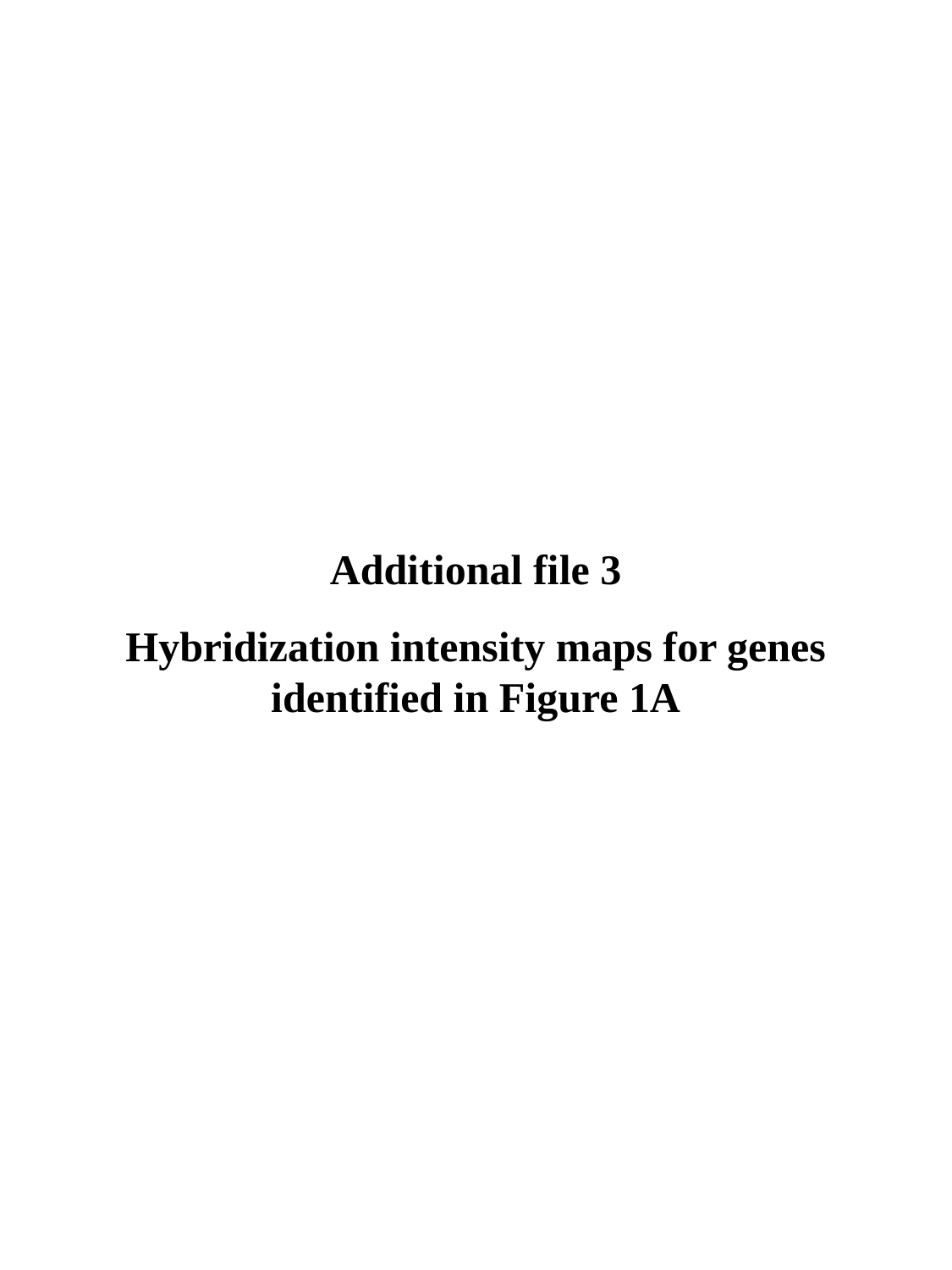

Additional file 3
Hybridization intensity maps for genes identified in Figure 1A

## Slide 2
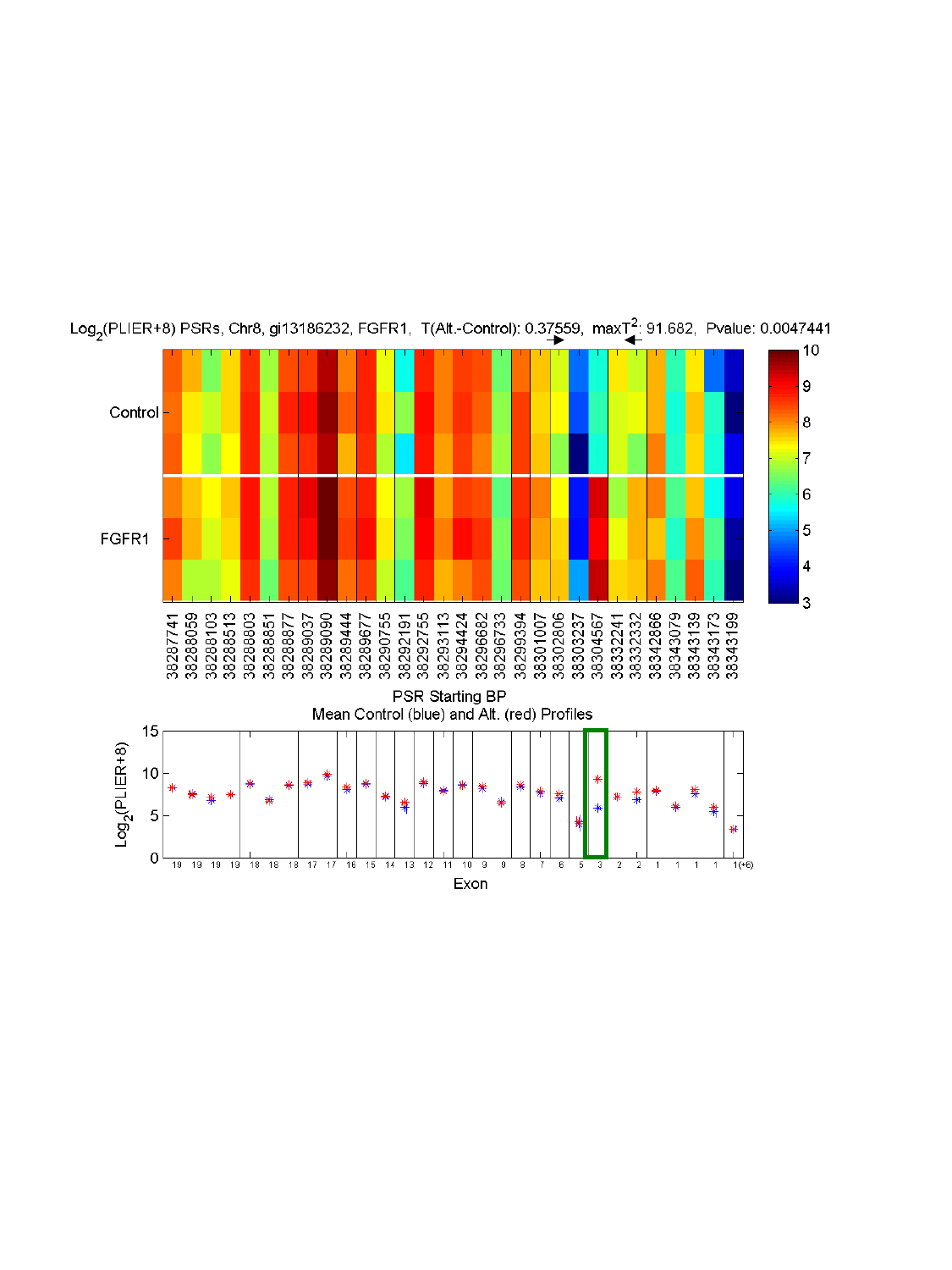

## Slide 3
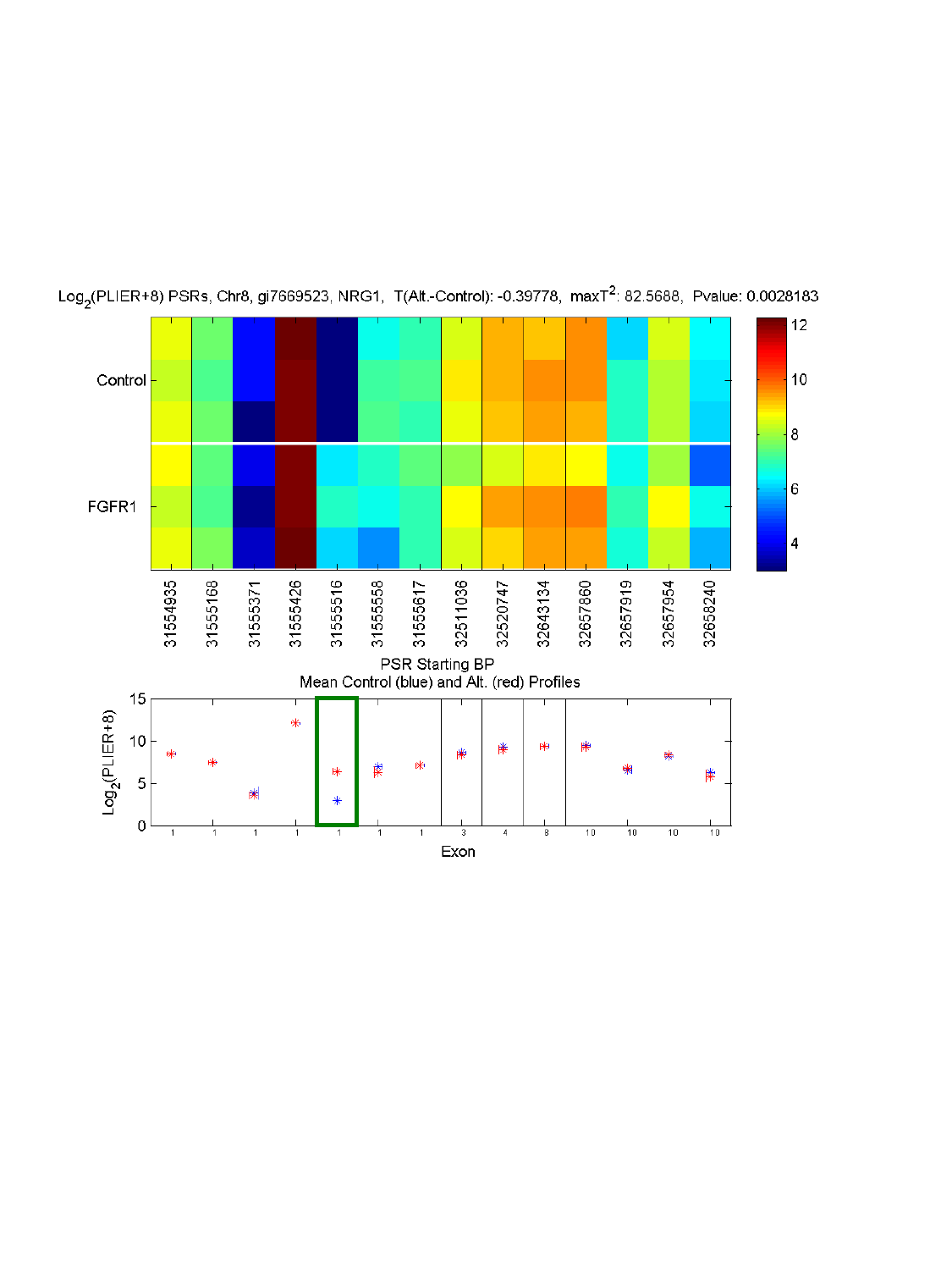

## Slide 4
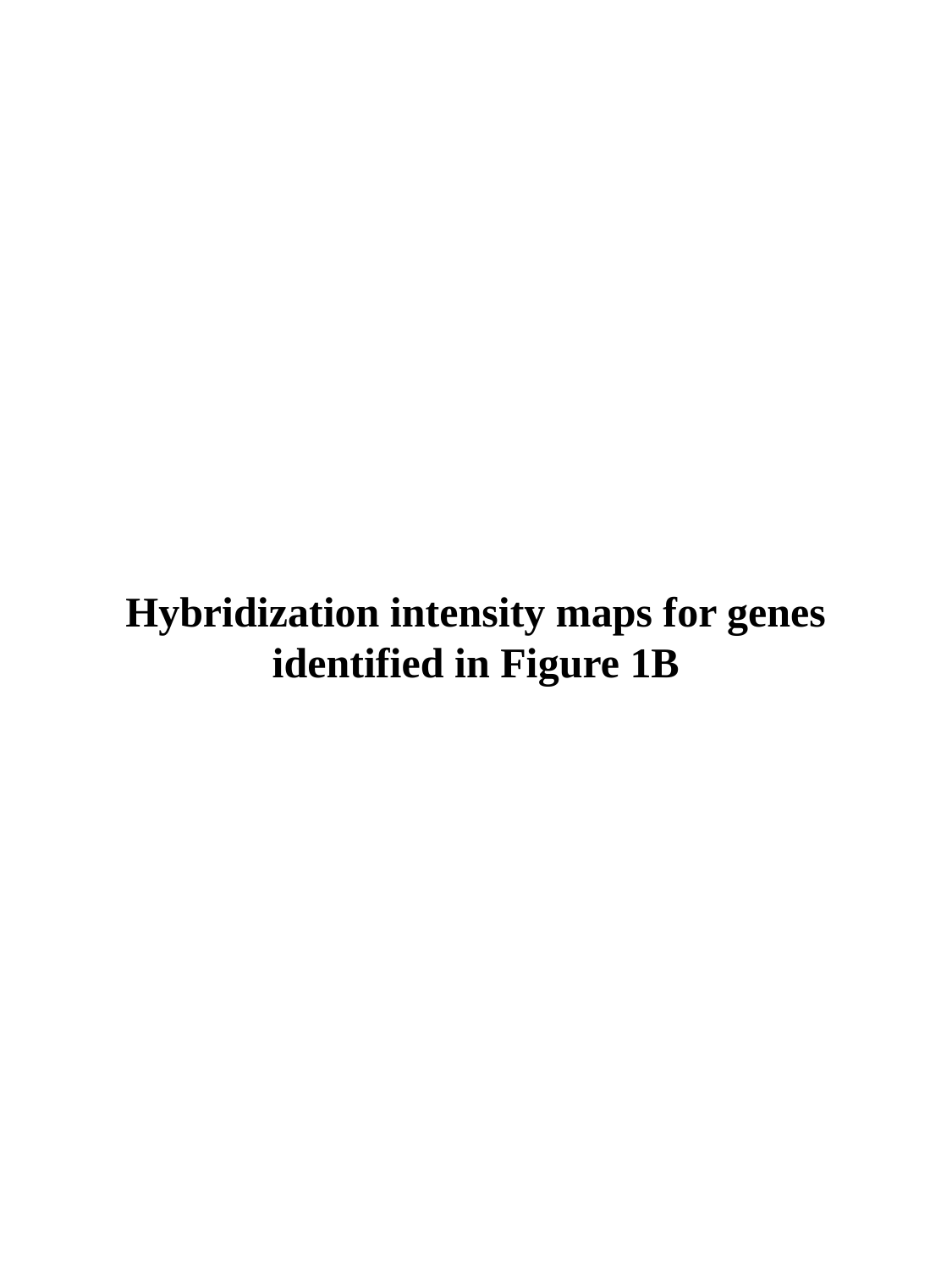

Hybridization intensity maps for genes identified in Figure 1B

## Slide 5
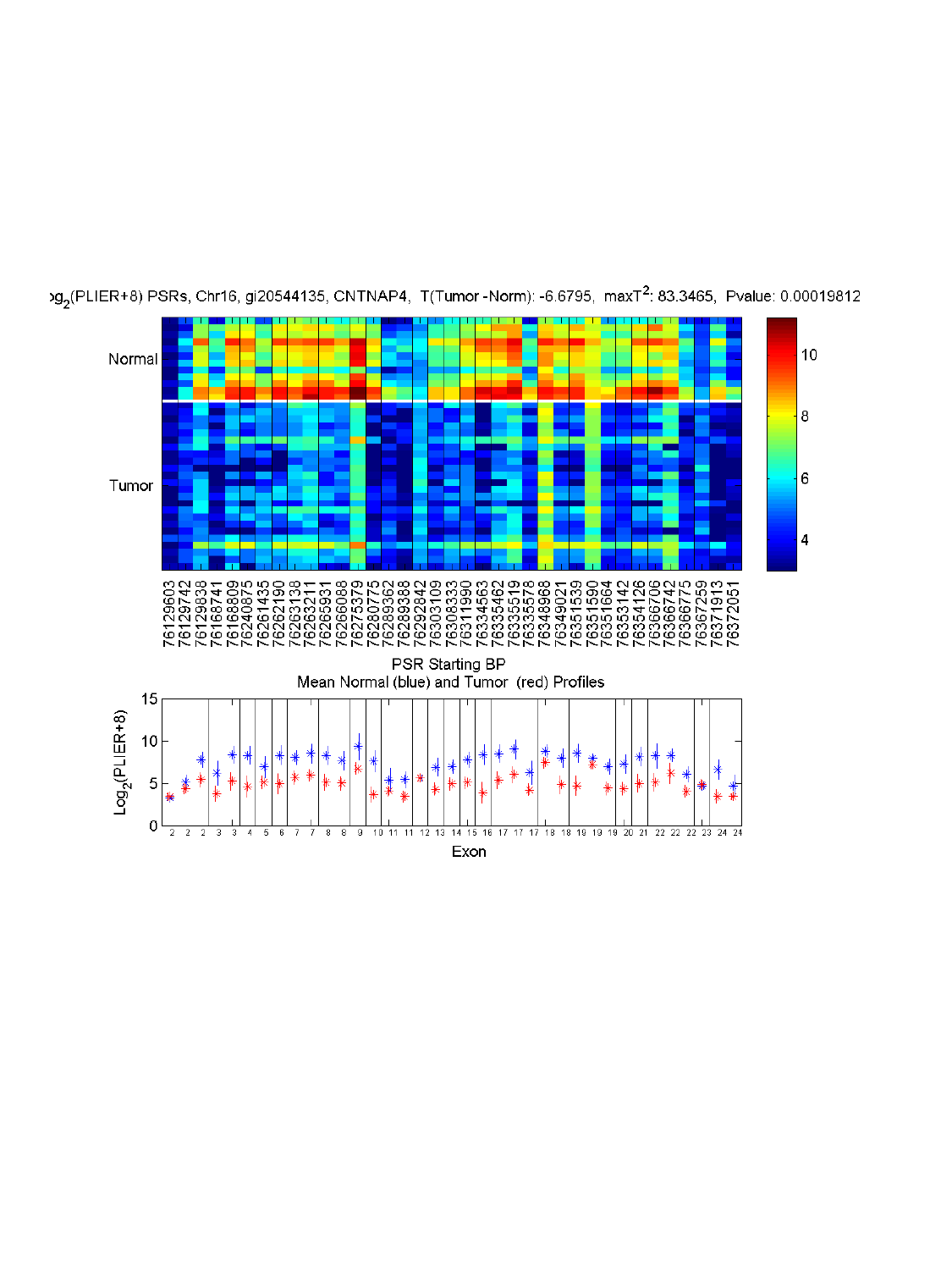

## Slide 6
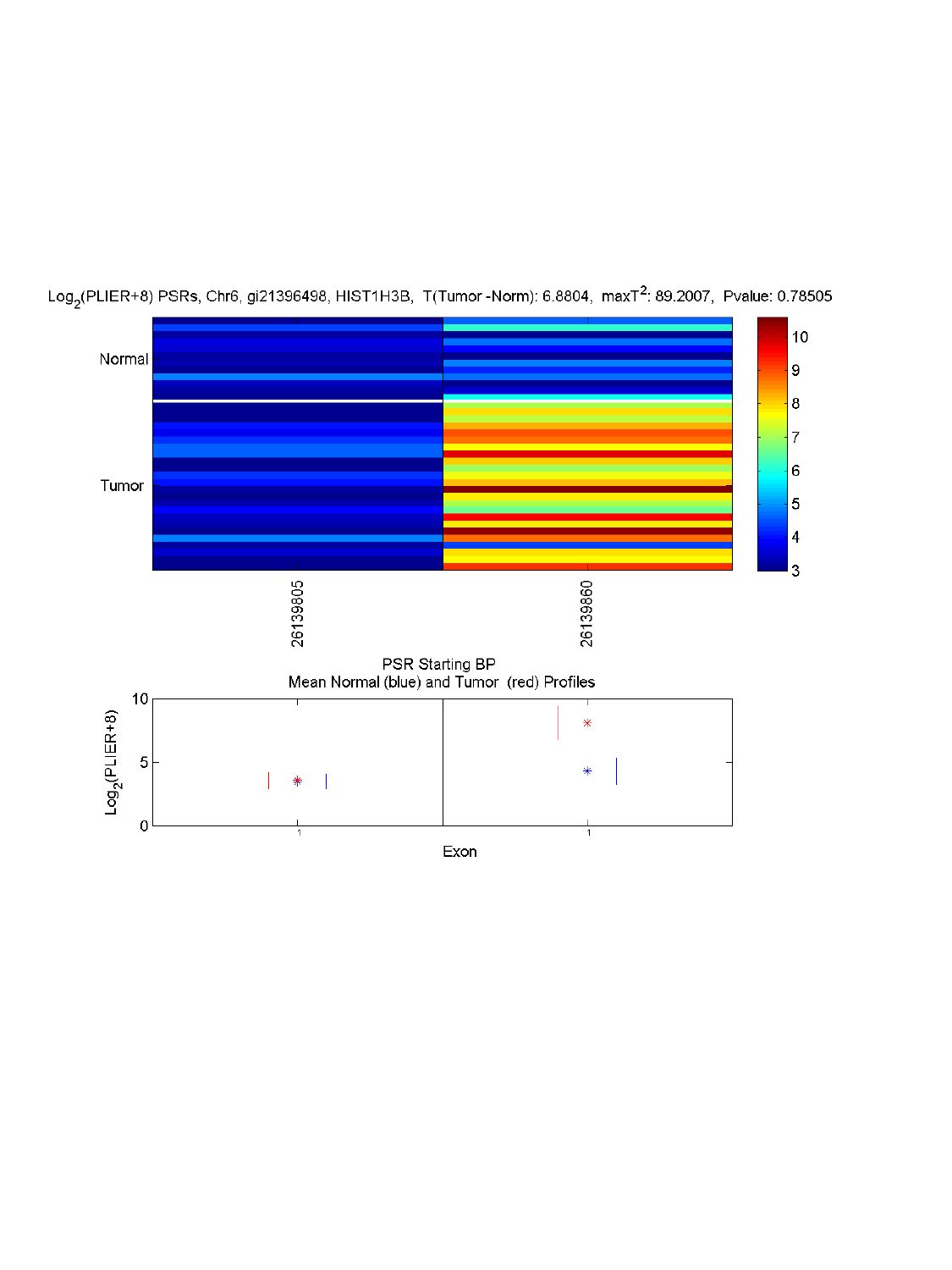

## Slide 7
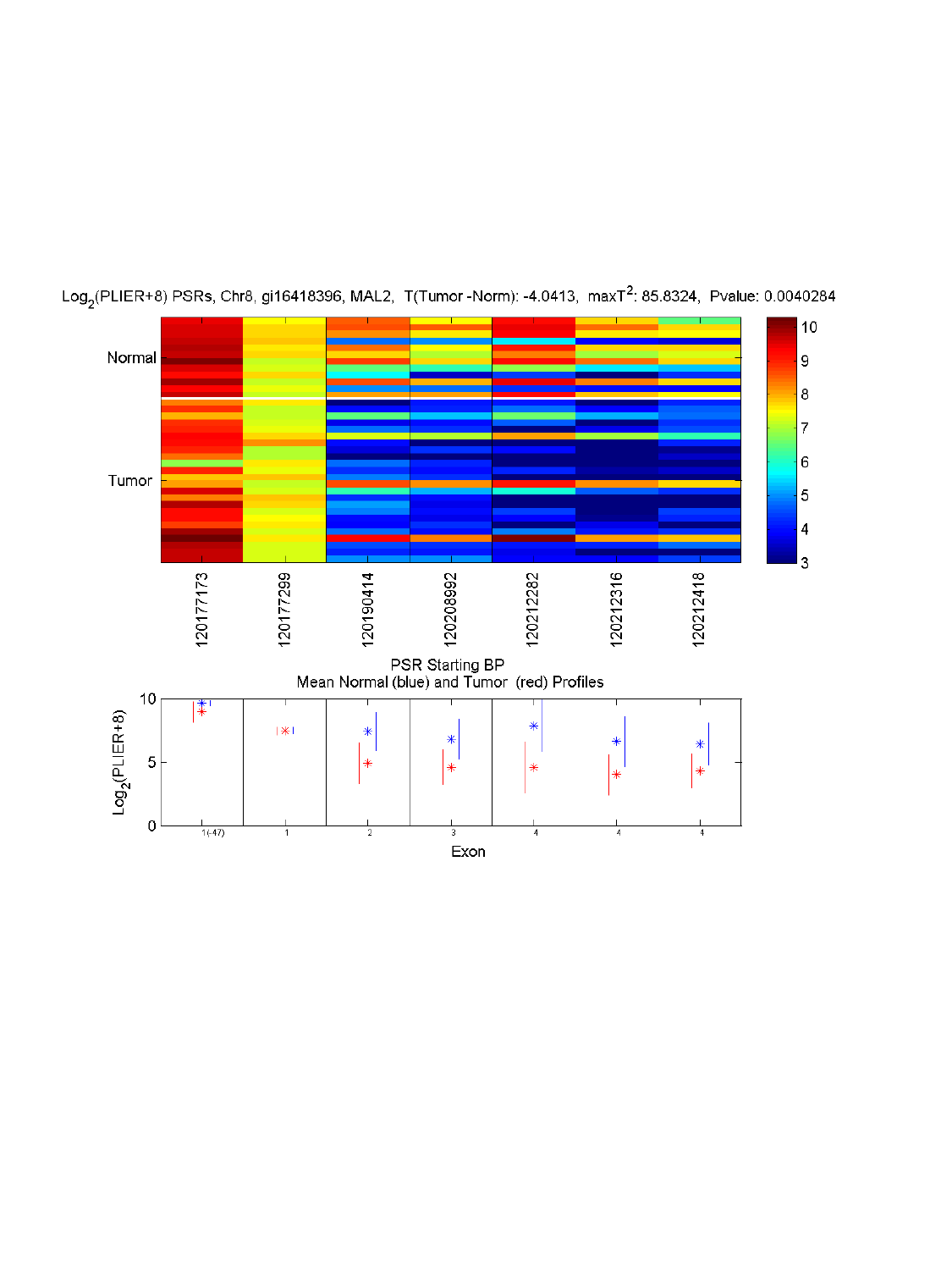

## Slide 8
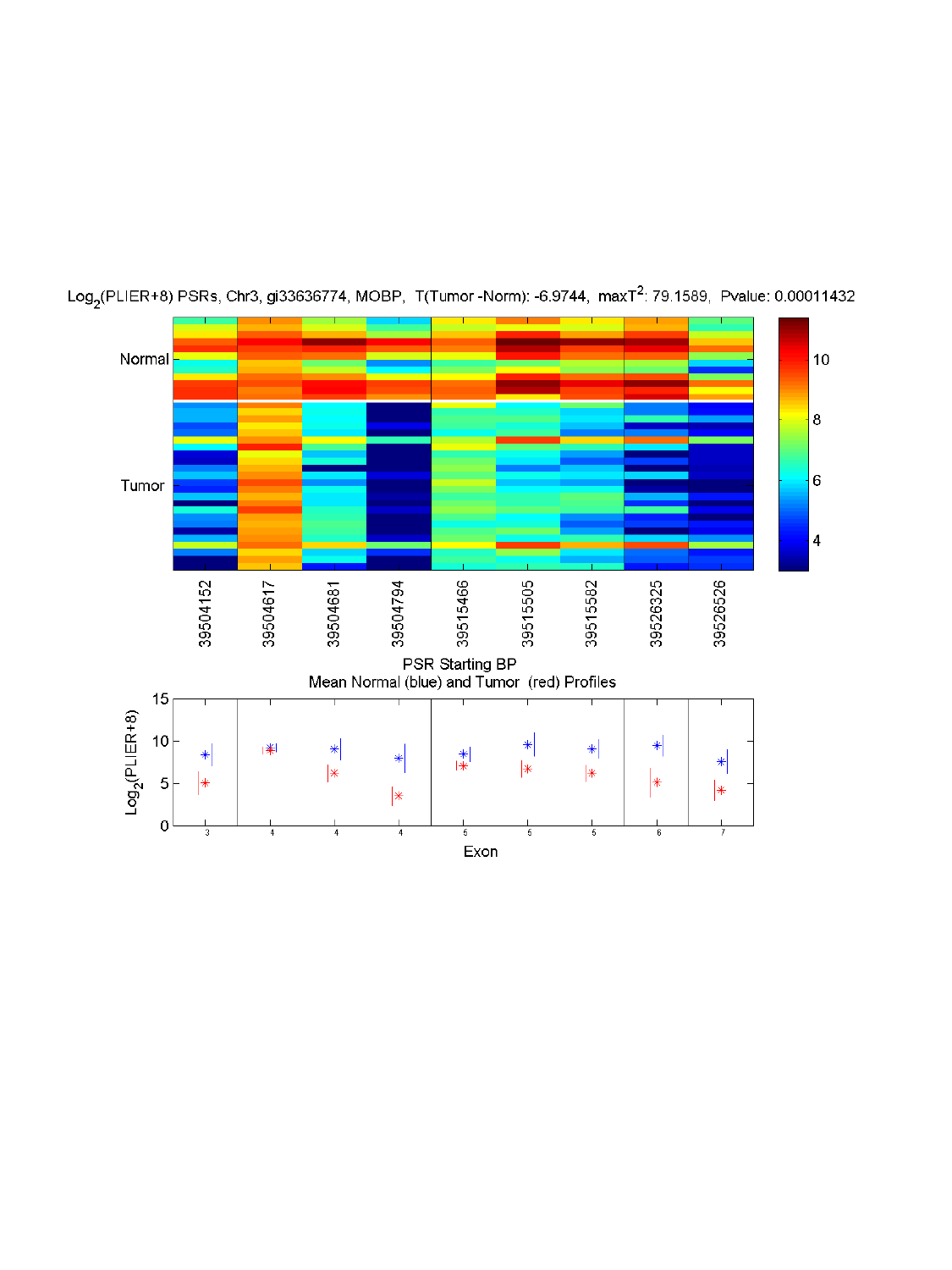

## Slide 9
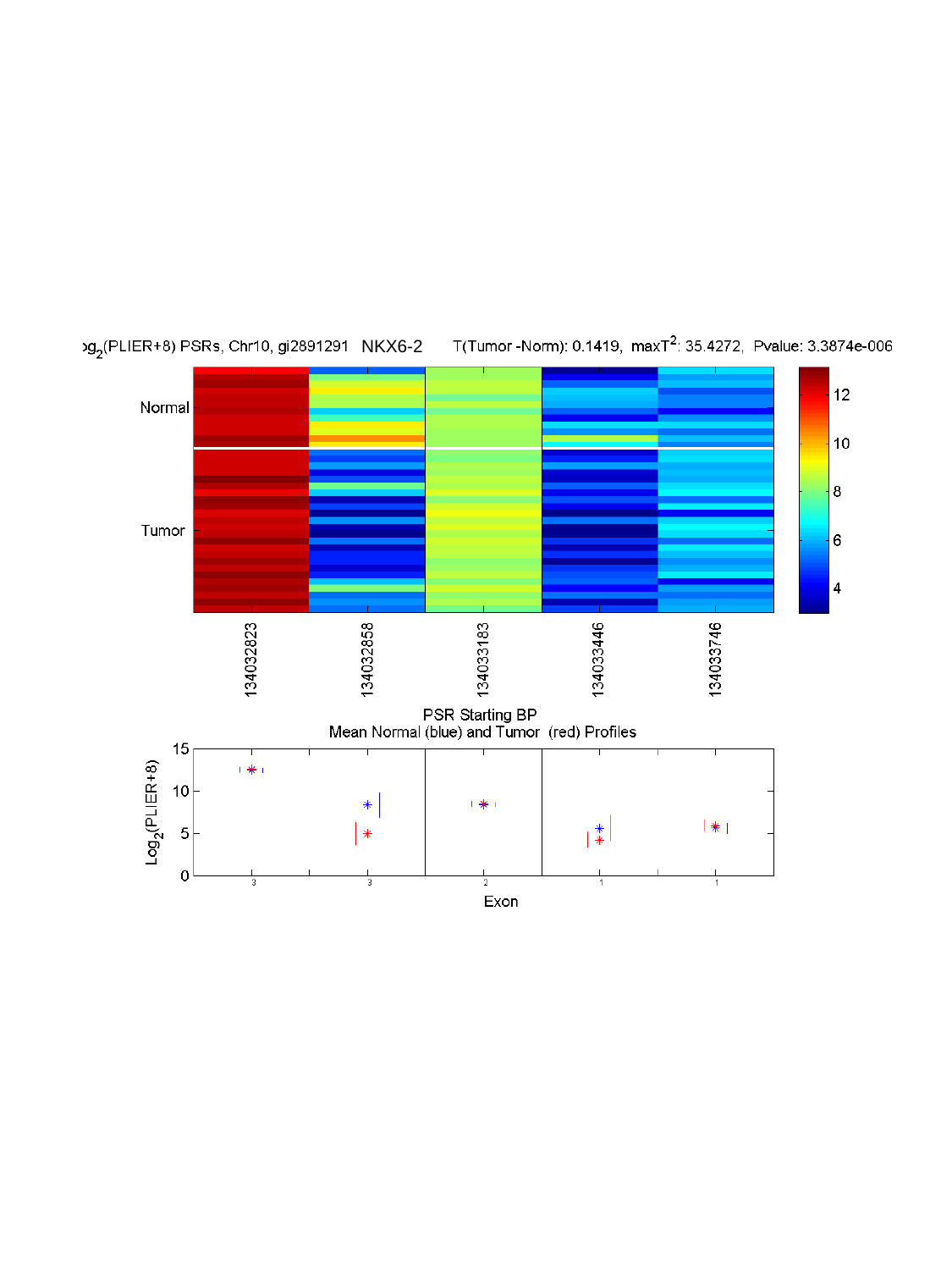

NKX6-2
