# Supplementary material for: Global analysis of aberrant pre-mRNA splicing in glioblastoma using exon expression arrays
Source: BMC Genomics. 2008 May 12;9:216. doi: 10.1186/1471-2164-9-216 (PMC2410136; doi:10.1186/1471-2164-9-216)

## Slide 1
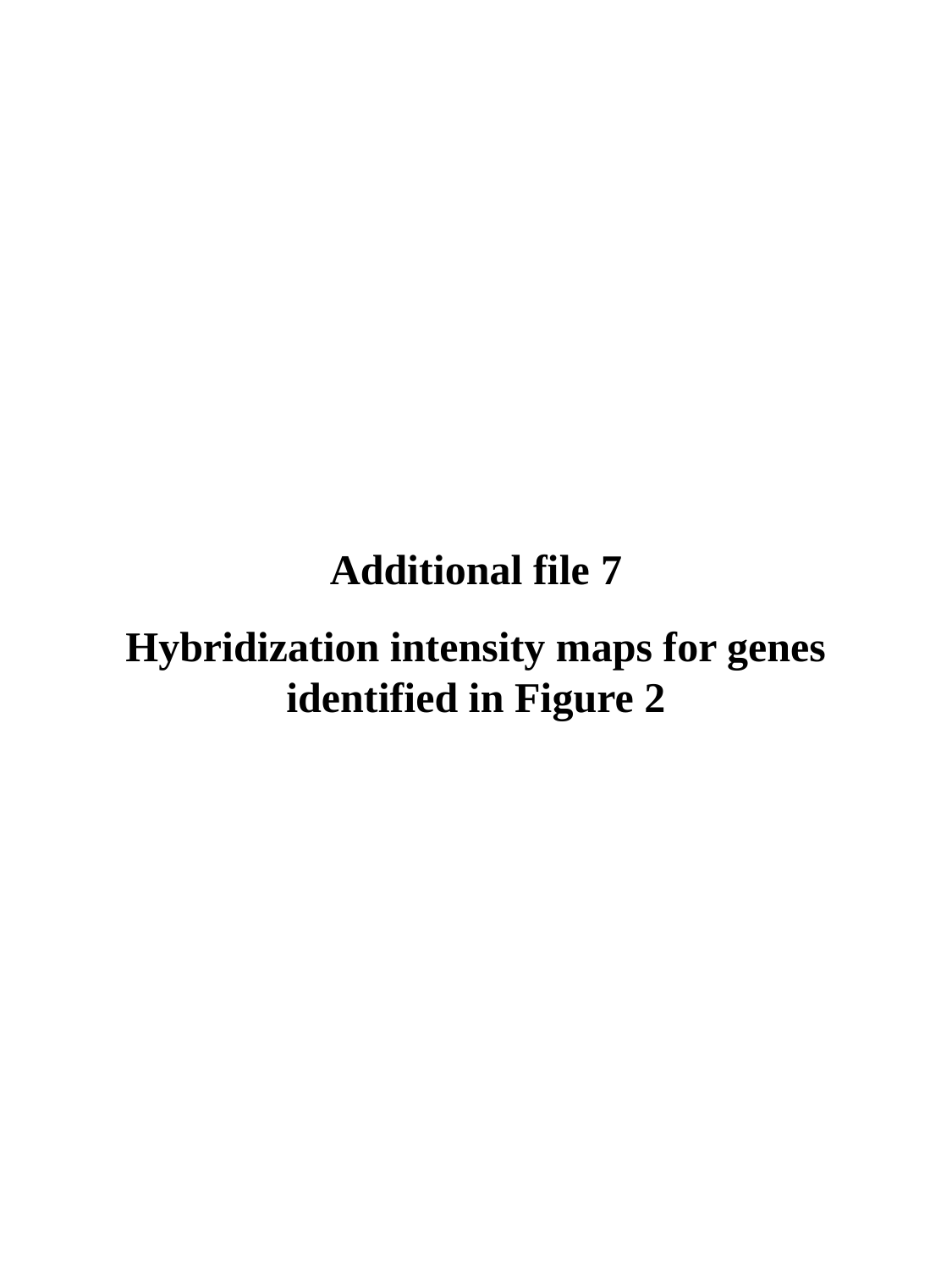

Additional file 7
Hybridization intensity maps for genes identified in Figure 2

## Slide 2
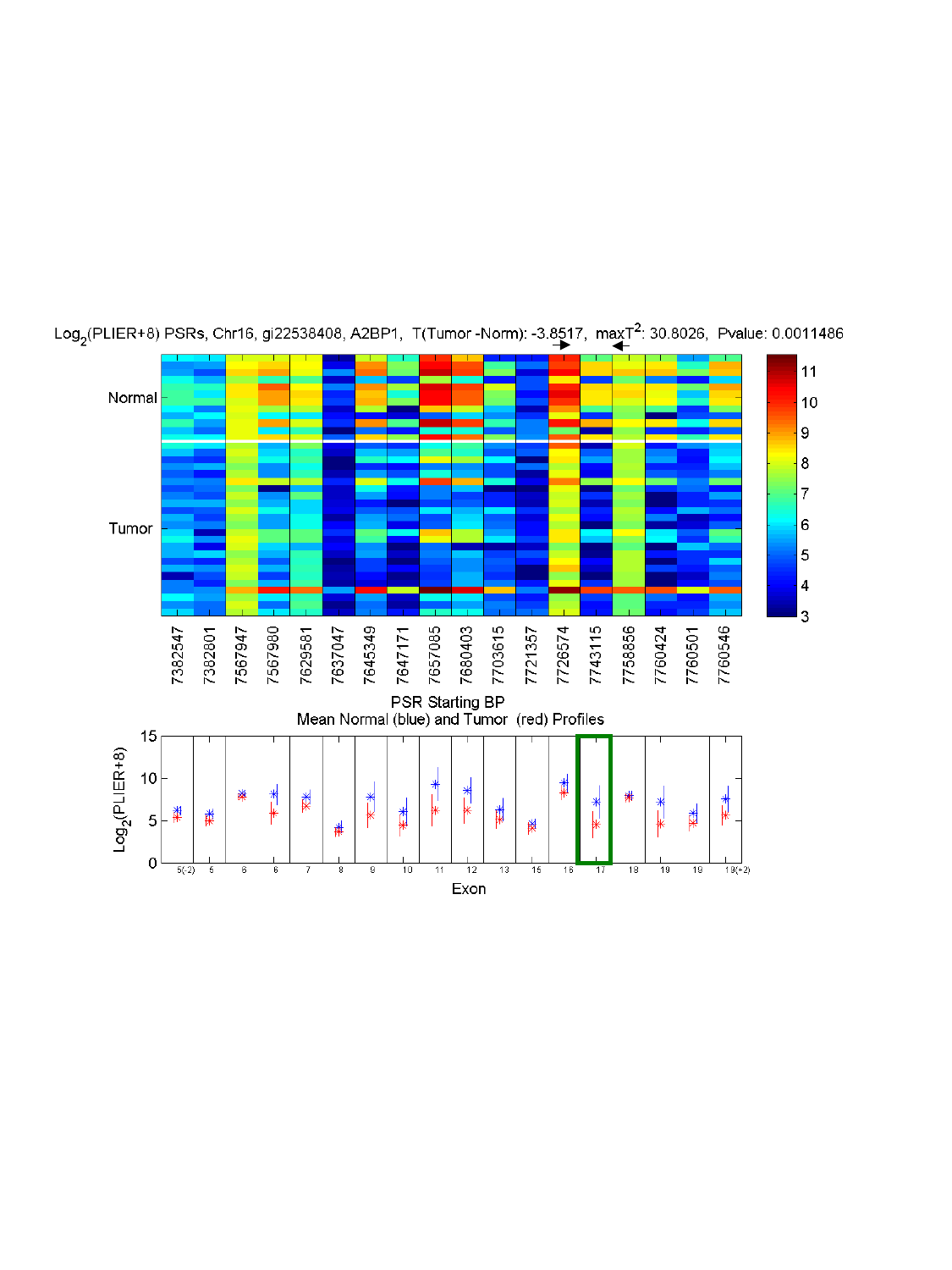

## Slide 3
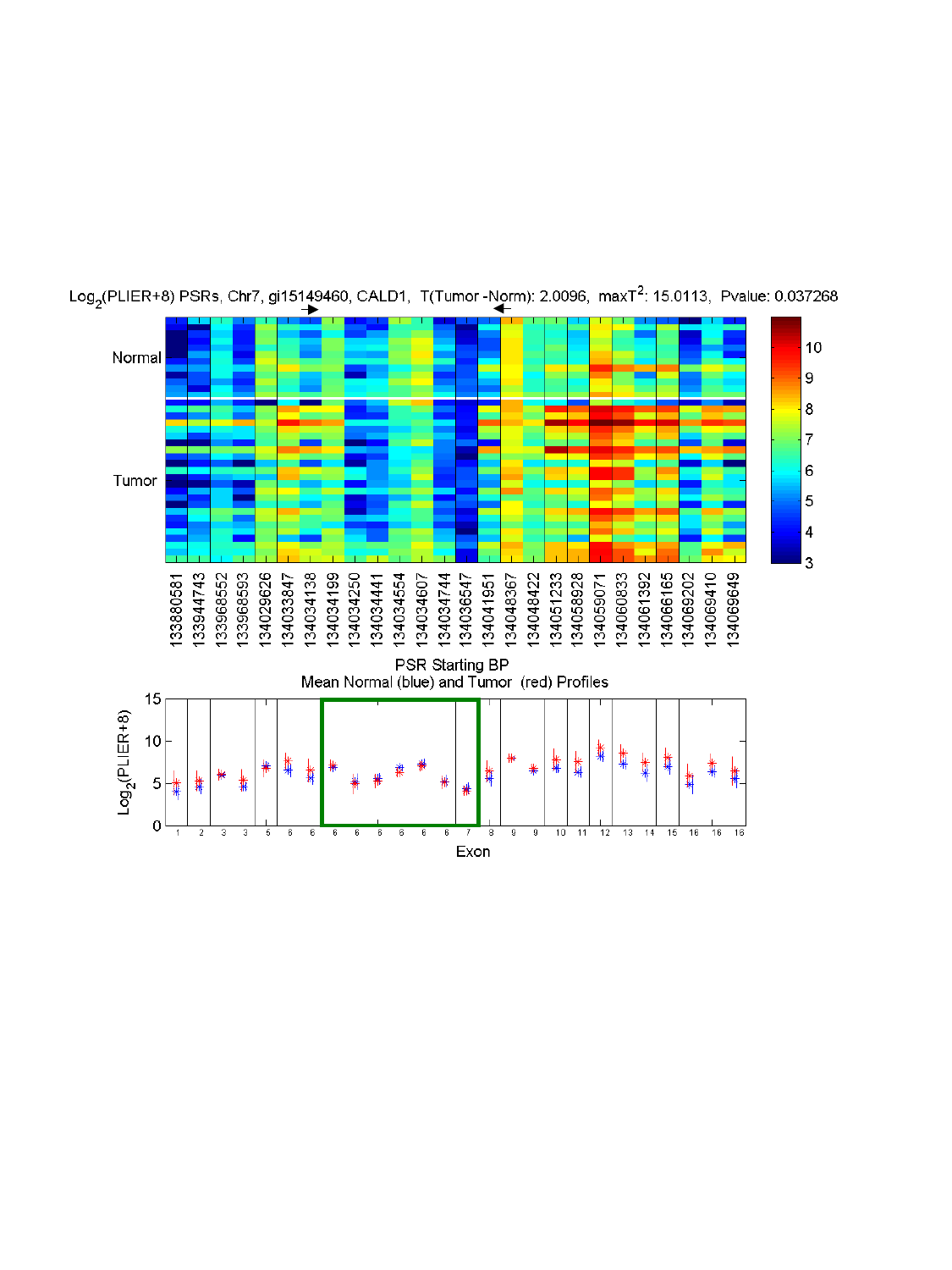

## Slide 4
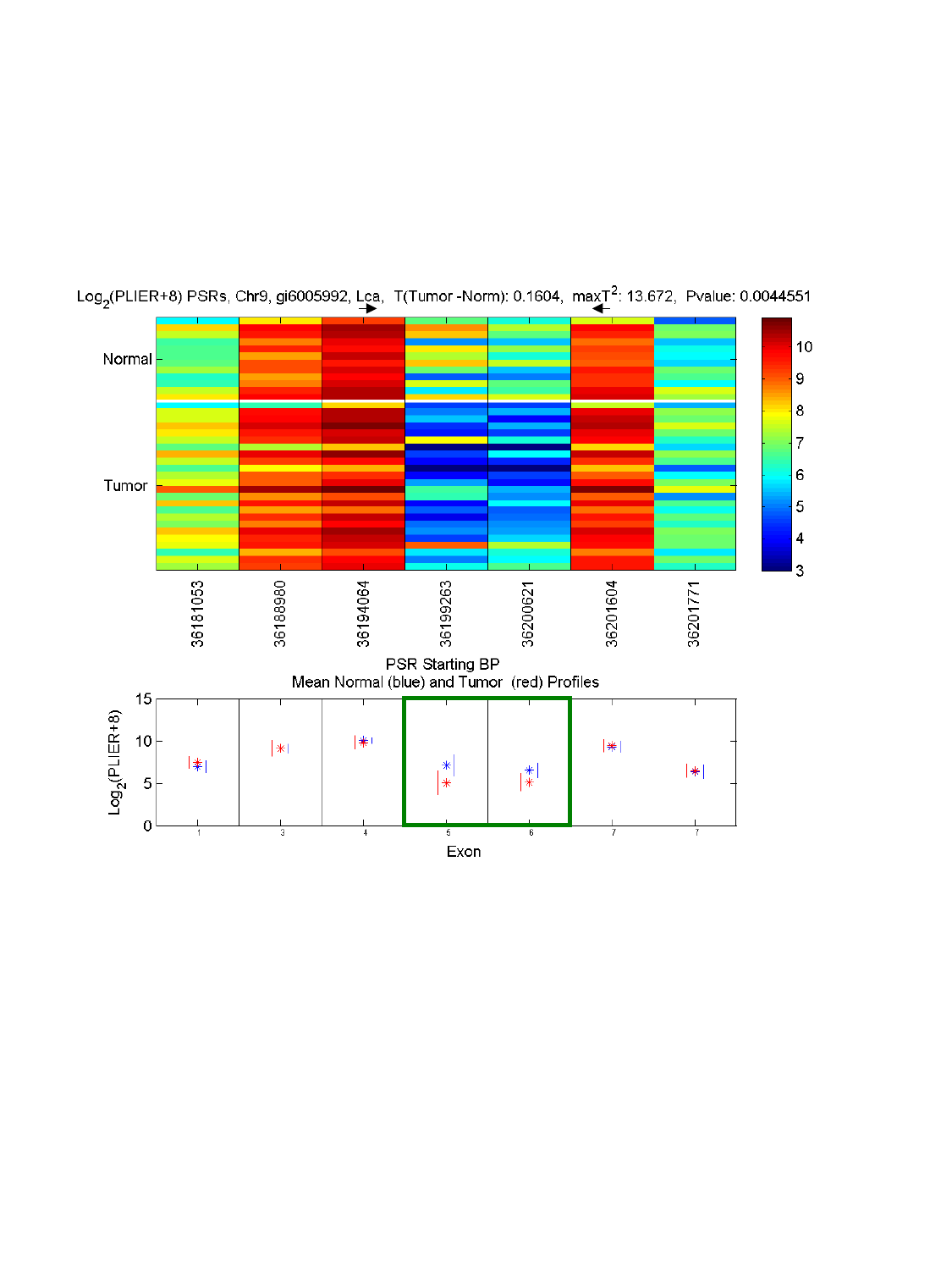

## Slide 5
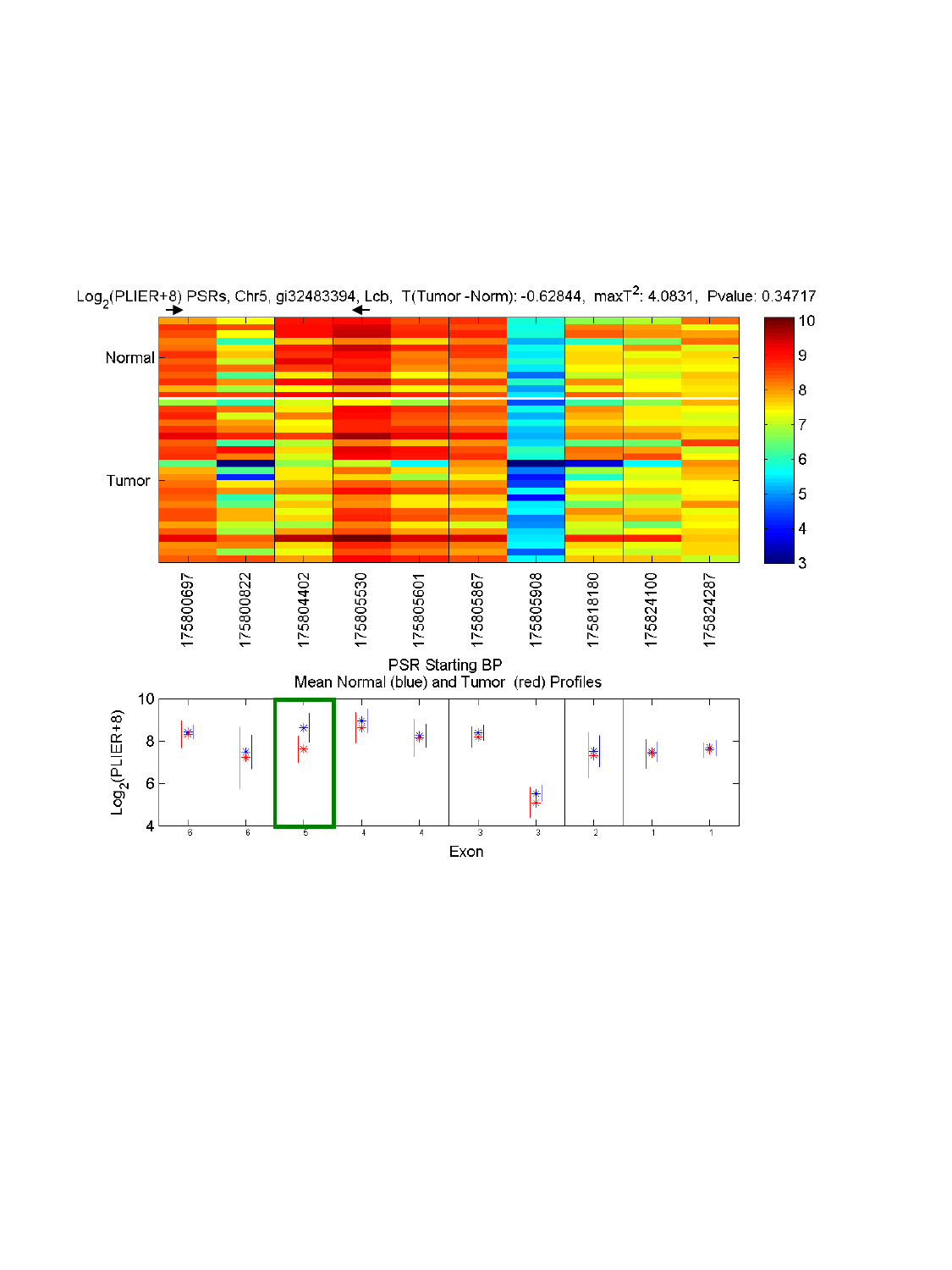

## Slide 6
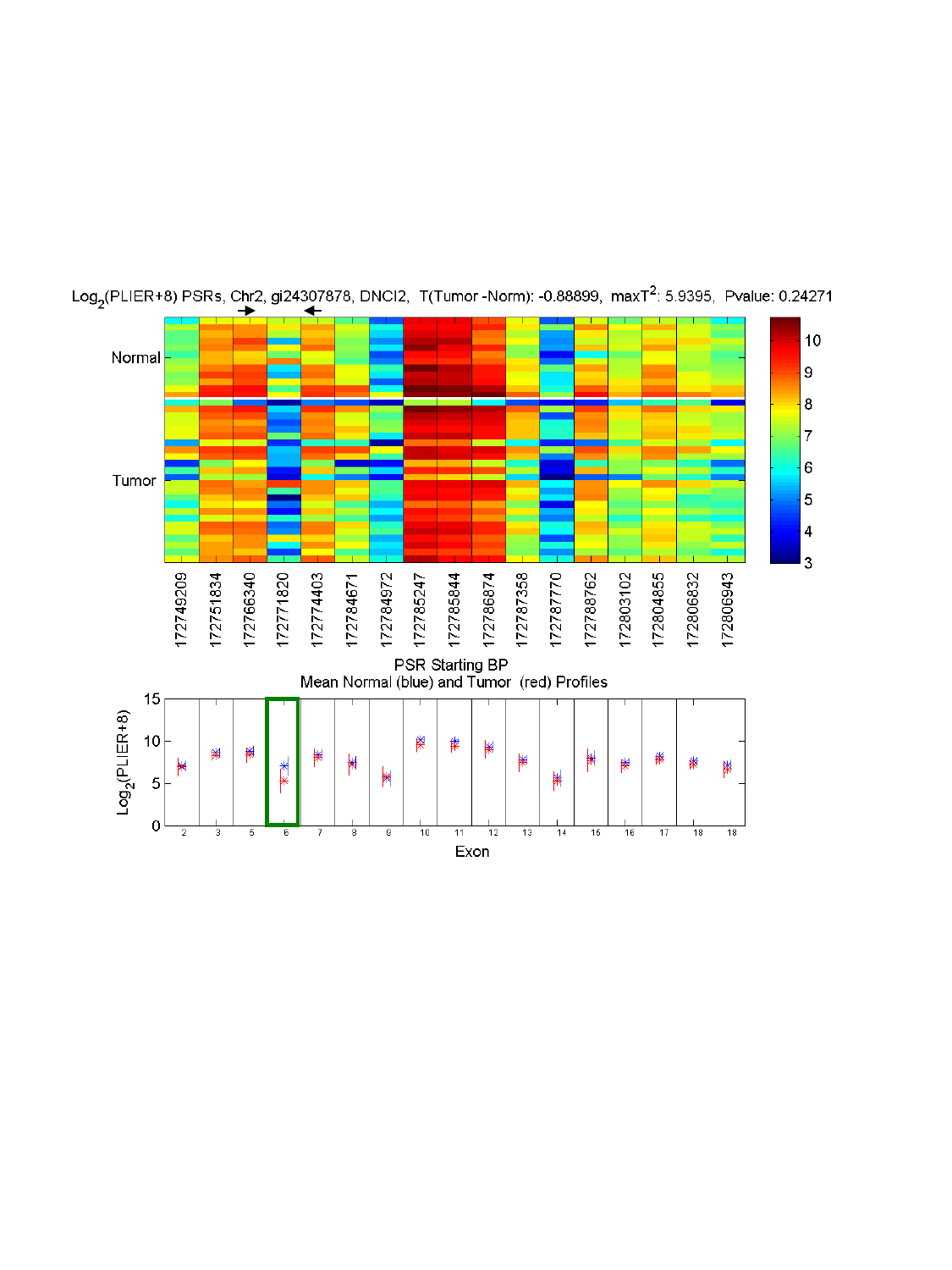

## Slide 7
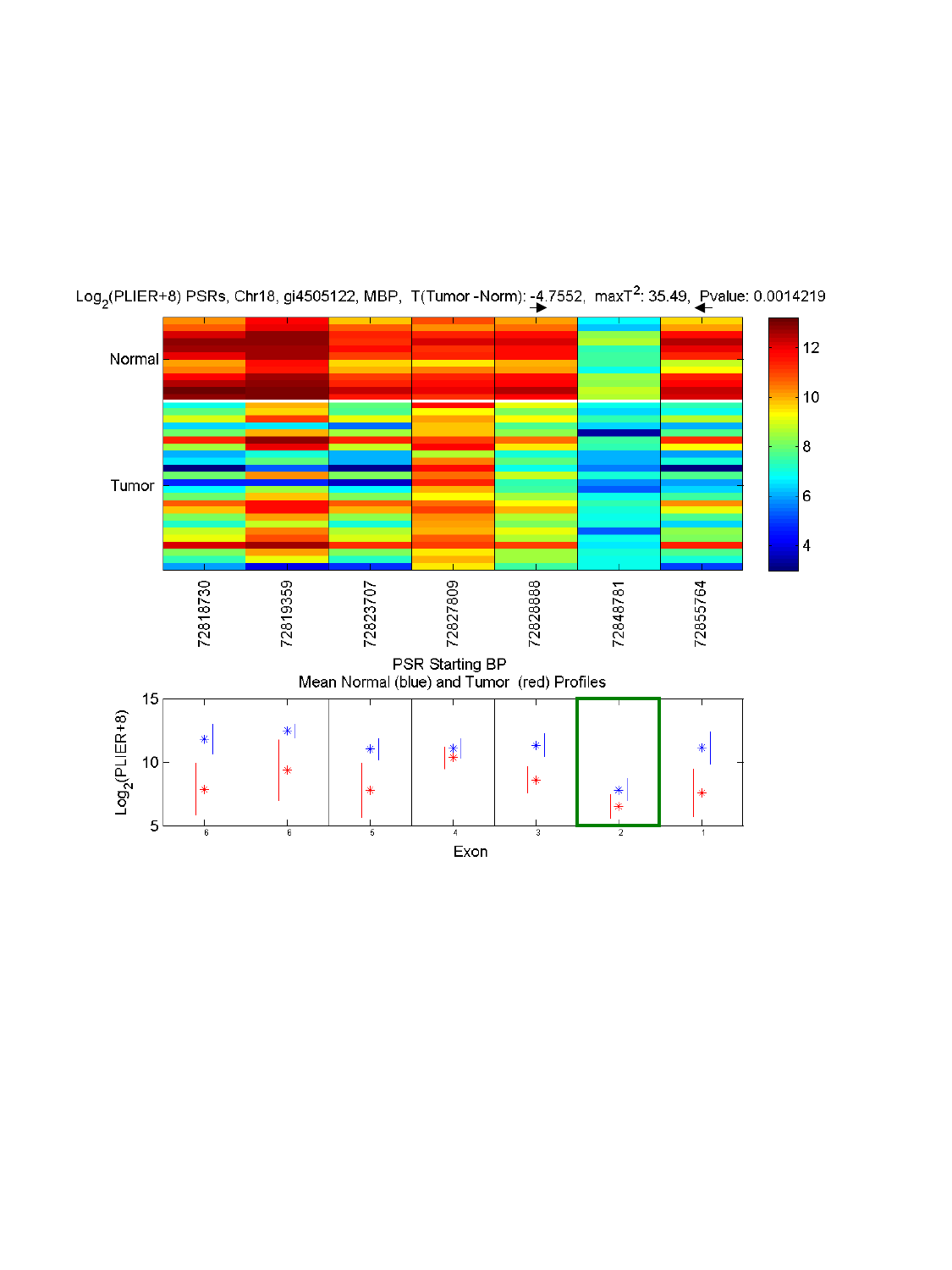

## Slide 8
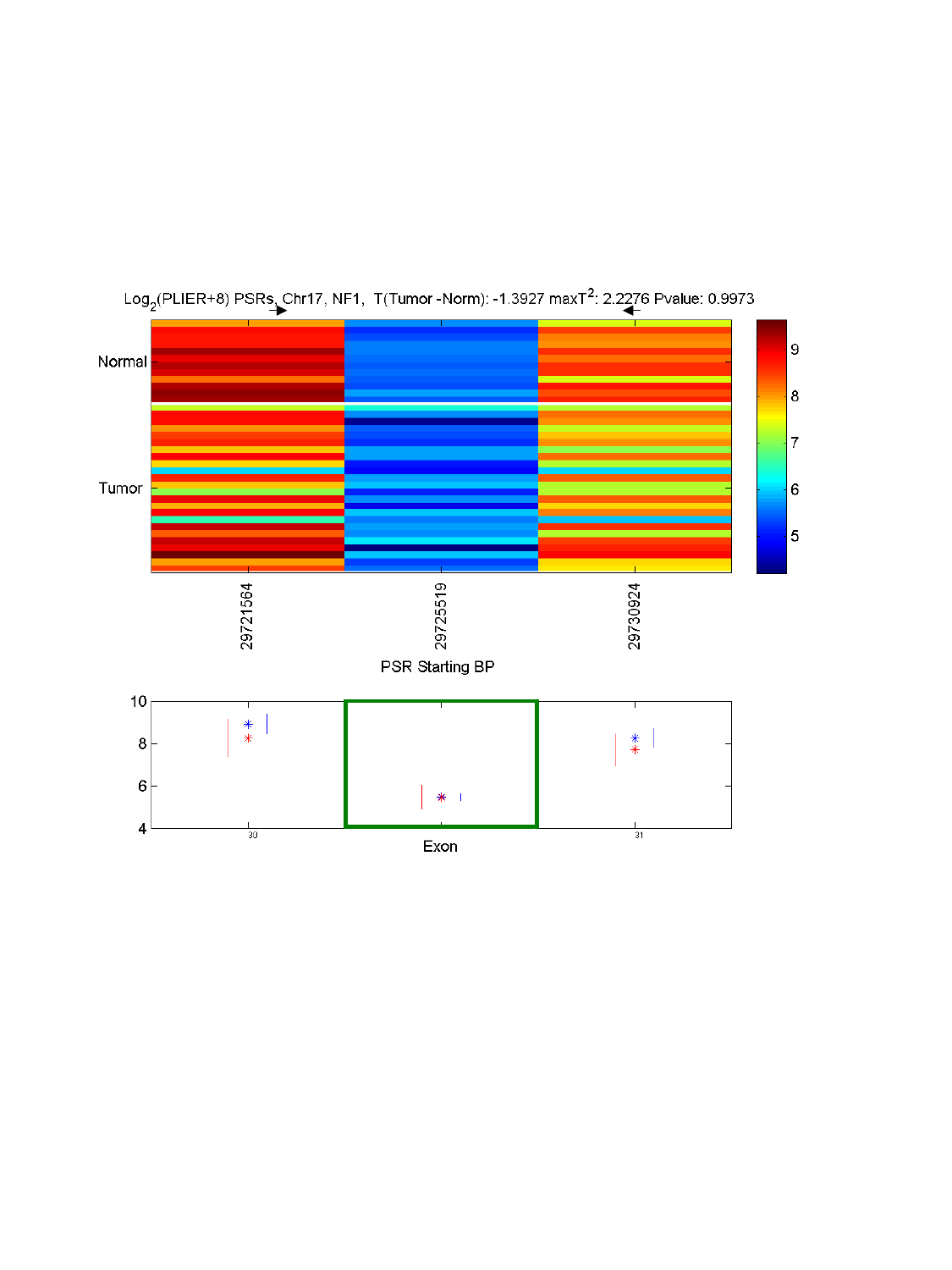

## Slide 9
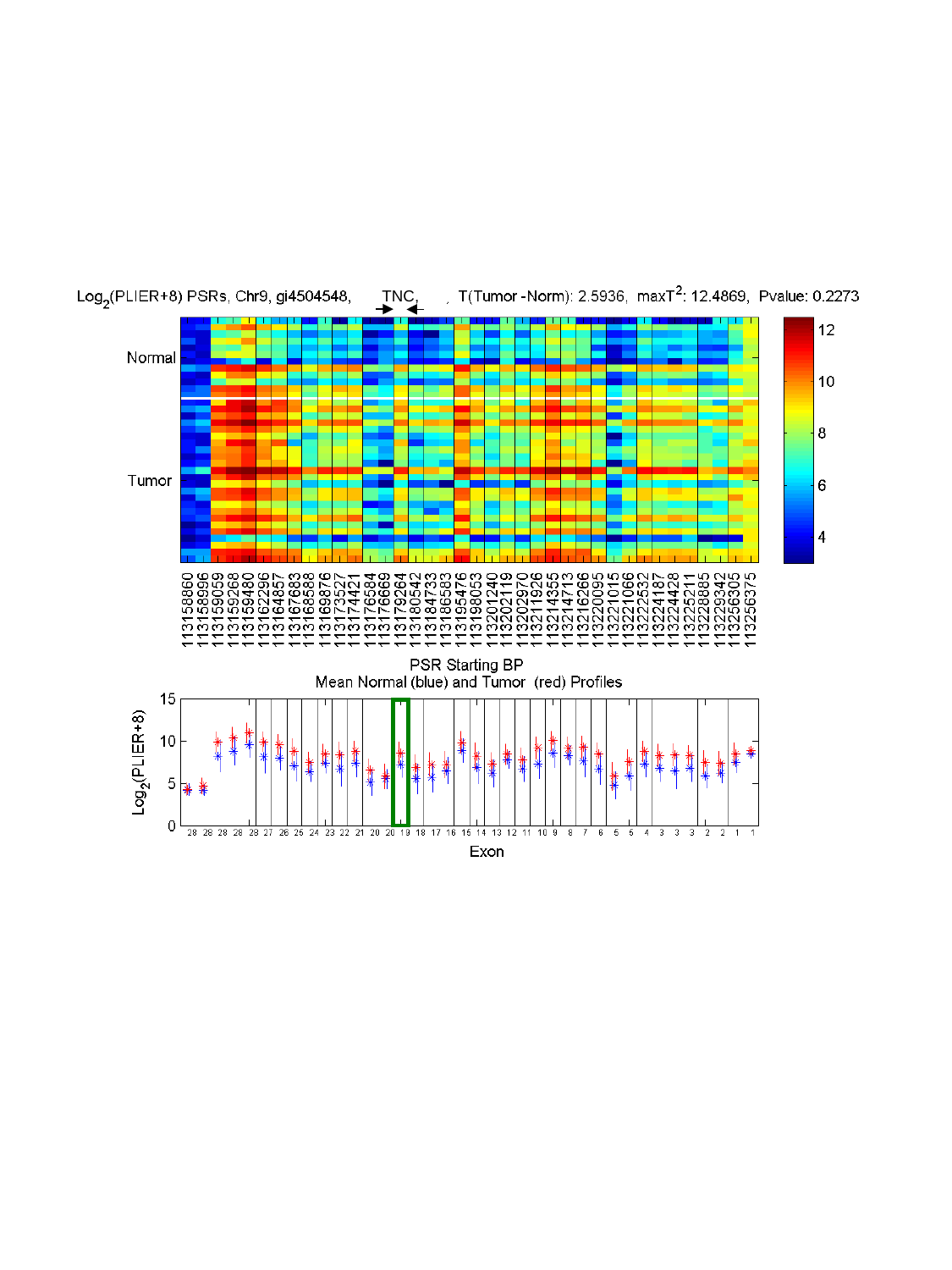

TNC,
TNC

## Slide 10
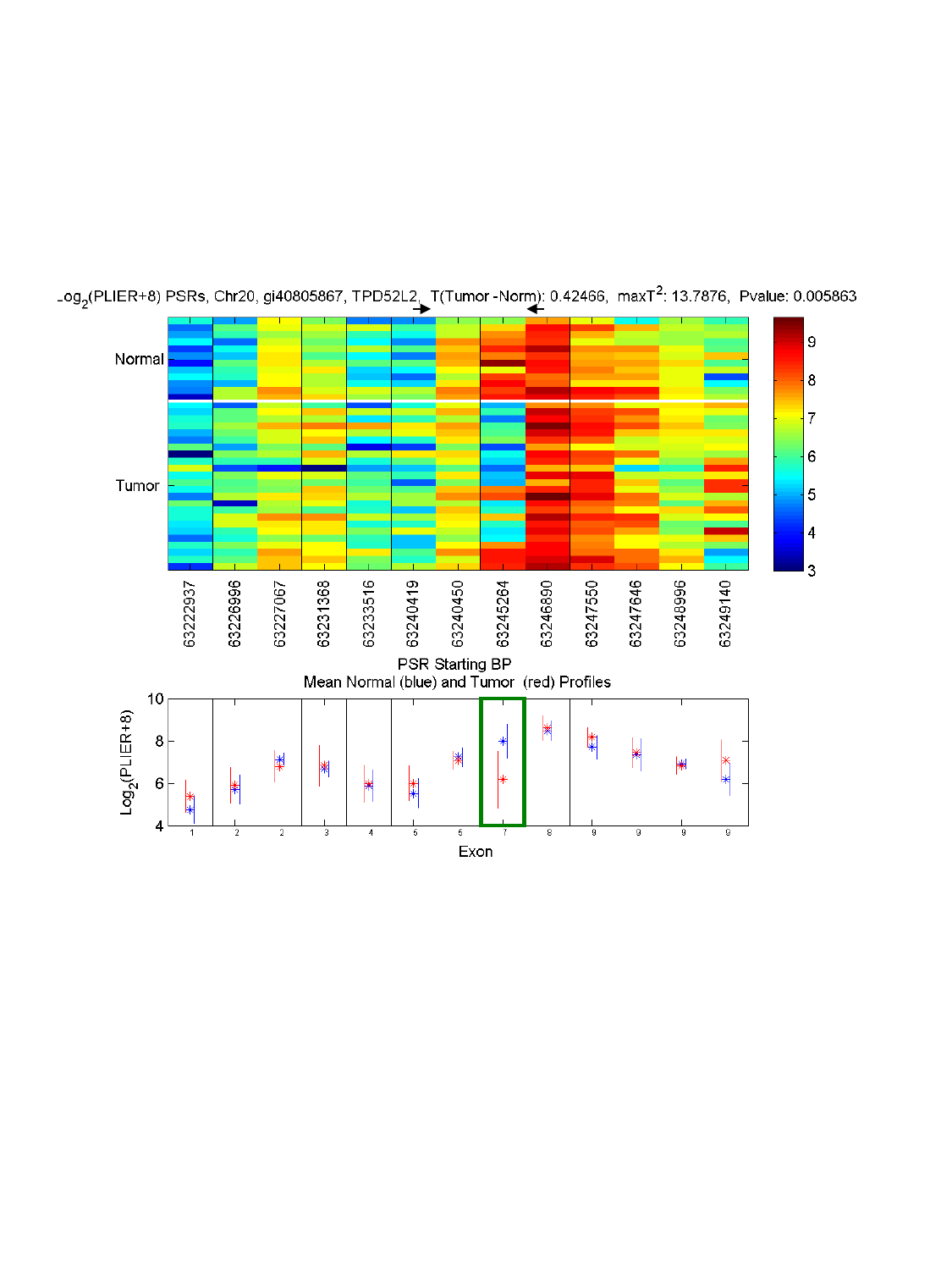

## Slide 11
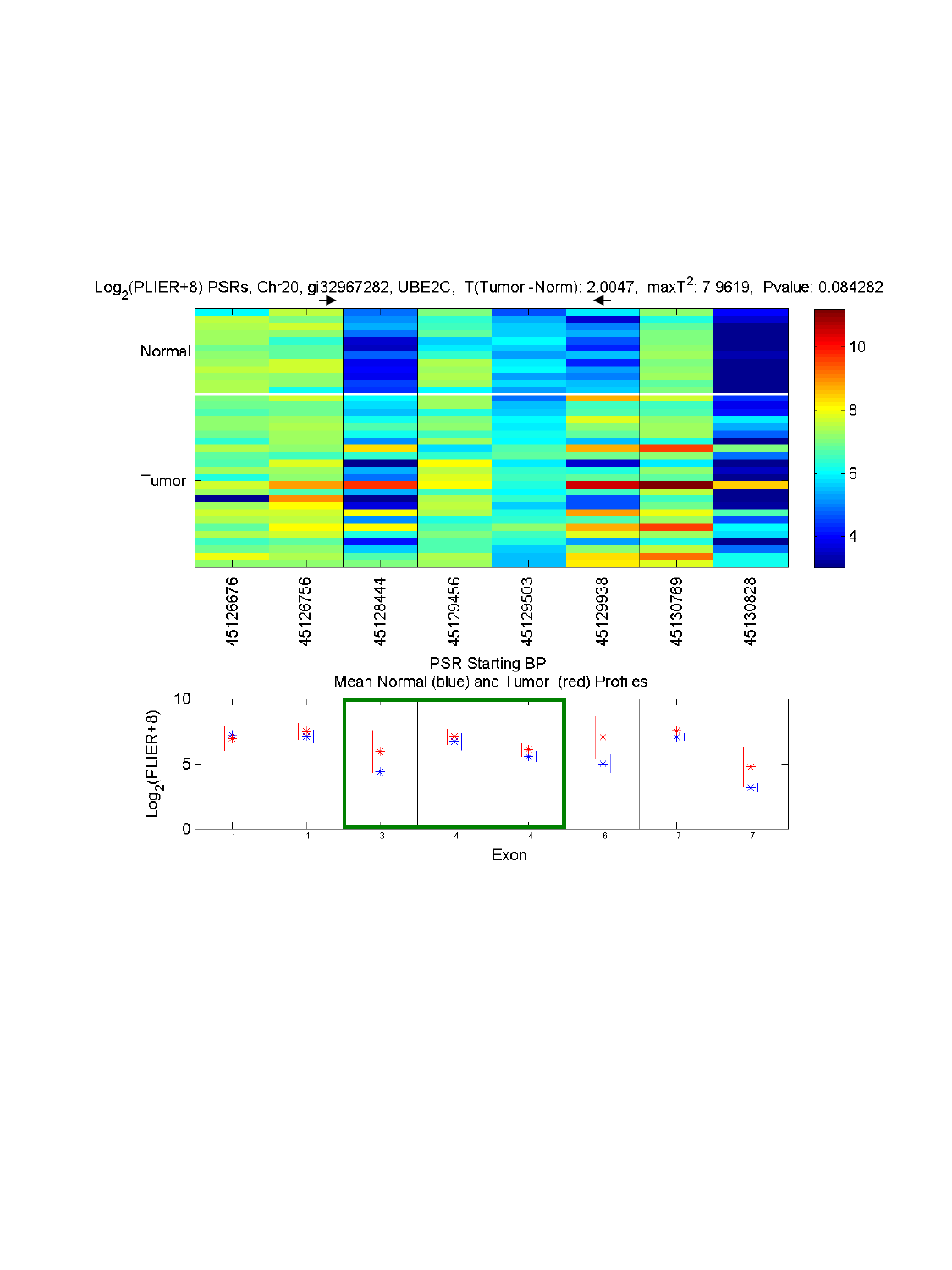

## Slide 12
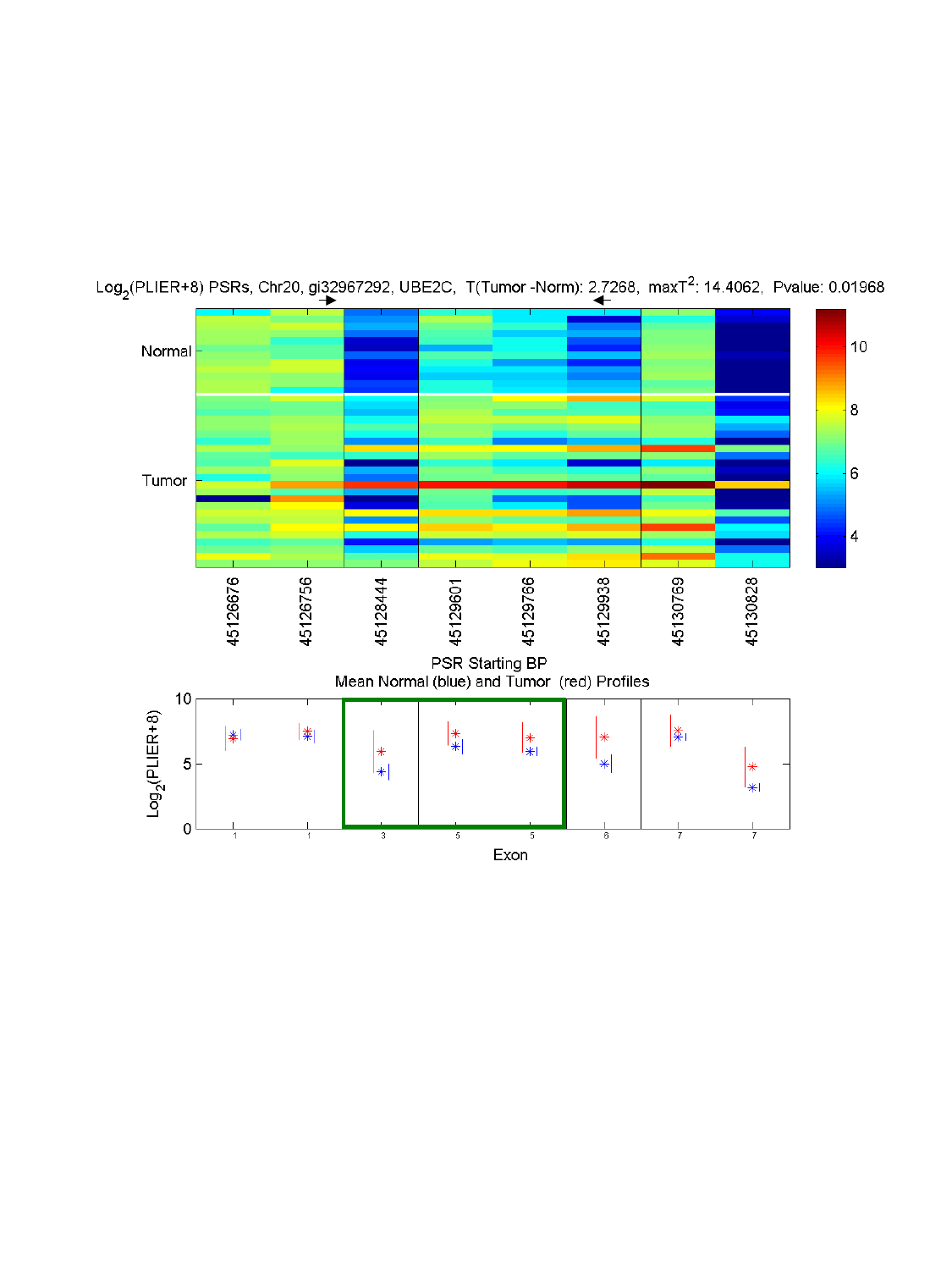

Supplement: Additional file 7 — Hybridization intensity maps for genes identified in Figure 2. Heat Maps for Figure 2. [file 1471-2164-9-216-S7.ppt]
